# Spatiotemporal endometrial transcriptome analysis revealed the luminal epithelium as key player during initial maternal recognition of pregnancy in the mare

**DOI:** 10.1038/s41598-021-01785-3

**Published:** 2021-11-16

**Authors:** Alba Rudolf Vegas, Giorgia Podico, Igor F. Canisso, Heinrich Bollwein, Carmen Almiñana, Stefan Bauersachs

**Affiliations:** 1 Institute of Veterinary Anatomy and Clinic of Reproductive Medicine, Department for Farm Animals, Vetsuisse Faculty Zurich, University of Zurich, Lindau, Switzerland; 2 Department of Veterinary Clinical Medicine, College of Veterinary Medicine, University of Illinois Urbana Champaign, Urbana, IL USA; 3 Clinic of Reproductive Medicine, Department for Farm Animals, Vetsuisse Faculty Zurich, University of Zurich, Lindau, Switzerland; 4 Institute of Veterinary Anatomy, Vetsuisse Faculty, University of Zurich, Lindau, Switzerland

**Keywords:** Transcriptomics, Reproductive biology, Embryology, Endocrinology, Animal physiology

## Abstract

During the period of maternal recognition of pregnancy (MRP) in the mare, the embryo needs to signal its presence to the endometrium to prevent regression of the corpus luteum and prepare for establishment of pregnancy. This is achieved by mechanical stimuli and release of various signaling molecules by the equine embryo while migrating through the uterus. We hypothesized that embryo’s signals induce changes in the endometrial gene expression in a highly cell type-specific manner. A spatiotemporal transcriptomics approach was applied combining laser capture microdissection and low-input-RNA sequencing of luminal and glandular epithelium (LE, GE), and stroma of biopsy samples collected from days 10–13 of pregnancy and the estrous cycle. Two comparisons were performed, samples derived from pregnancies with conceptuses ≥ 8 mm in diameter (comparison 1) and conceptuses ≤ 8 mm (comparison 2) versus samples from cyclic controls. The majority of gene expression changes was identified in LE and much lower numbers of differentially expressed genes (DEGs) in GE and stroma. While 1253 DEGs were found for LE in comparison 1, only 248 were found in comparison 2. Data mining mainly focused on DEGs in LE and revealed regulation of genes related to prostaglandin transport, metabolism, and signaling, as well as transcription factor families that could be involved in MRP. In comparison to other mammalian species, differences in regulation of genes involved in epithelial barrier formation and conceptus attachment and implantation reflected the unique features of equine reproduction at the time of MRP at the molecular level.

## Introduction

Maternal recognition of pregnancy (MRP) is a complex process that is not fully understood in the horse. It refers to the production of signals by the conceptus (embryo and its associated membranes) that eventually act on the corpus luteum (CL) to ensure its maintenance and progesterone (P4) production^1^. Since pregnancy depends mainly on P4 during the first month, MRP signals have to prevent the endometrium from secreting luteolytic prostaglandin F_2_ alpha (PGF2a), and thus regression of the CL^2^. However, the specific embryonic signals of MRP are still unknown in the mare^2^.

Pregnancy in the mare is characterized by several unique features. One of the most intriguing features is probably the acellular glycoprotein capsule surrounding the blastocyst after entering the uterus around day 6 until day 23^3^. This capsule is vital for embryo survival during the first weeks of pregnancy^4^ and protects the embryo during the migration phase in the uterus driven by uterine contractions until around day 16 after ovulation^5^.

It has been suggested that MRP is achieved by a combination of mechanical stimuli and release of signaling molecules by the migrating equine embryo since restriction of mobility resulted in failure in MRP^6^. Among the variety of molecules secreted during this migration phase by the conceptus, are estrogens^7^. However, estrogens seem not to be the embryonic signal for MRP in the mare^8^. A study of conceptus steroid production revealed 17 alpha-hydroxyprogesterone as the major metabolite but the effects on the endometrium are unknown^9^. In addition, prostaglandin E2 (PGE2) and PGF2a production in equine conceptuses has been shown, but the involvement in MRP remained unclear^10^.

With the advent of new techniques for comprehensive transcriptome analysis, several studies have been performed to analyze endometrial gene expression changes in response to the presence of a conceptus during the period of MRP in the mare, mainly focusing on days 8, 12, 13, 13.5 or 16^11–16^. Two studies analyzed endometrial miRNA expression during MRP in addition to mRNAs^13,16^, and Smits et al.^17^ performed also a comparison to gene expression in the embryo and protein expression in the uterine fluid. At day 8 after ovulation, Merkl et al.^11^ did not identify changes in endometrial gene expression of pregnant versus non-pregnant mares, but changes were evident on day 12. More pronounced differences in pregnant samples to cyclic controls were found on day 16 than on Day 12^18,19^. Many of the obtained differentially expressed genes (DEGs) in the different studies were related to steroid hormone and prostaglandin (PG) regulation, angiogenesis, regulation of decidualization and implantation, and vascular remodeling. Examples of genes consistently found as upregulated in endometrium of pregnant mares and suggested to play a role in establishment of pregnancy are insulin like growth factor binding protein 1 (*IGFBP1*), *IGFBP3*, fibroblast growth factor 9 (*FGF9*), crystallin alpha B (*CRYAB*), GM2 ganglioside activator (*GM2A*), solute carrier family 36 A (*SLC36A*), amphiregulin (*AREG*), ERBB receptor feedback inhibitor 1 (*ERRFI1*), prostaglandin E receptor 4 (*PTGER4*), and prostaglandin reductase 1 (*PTGR1*). However, despite of the wealth of endometrial gene expression data, no conclusions could be drawn on the specific nature of the embryonic recognition signal.

The main limitation of the previous studies of the equine endometrium could be the use of whole tissue samples. Our previous studies of cell type composition of equine endometrial biopsy samples and cell type-specific endometrial gene expression analyses in the pig^11,20,21^ indicated a strong influence of cellular composition of the biopsy on the results and highly cell type-specific differential gene expression (DGE). In a recent study, our group used an approach combining laser capture microdissection (LCM) and low-input RNA-sequencing for the analysis of endometrial biopsies from pregnant and cyclic mares on Day 12 to study if DGE towards embryo signaling was cell type-specific^22^. Despite the limitations of low RNA quality of the obtained samples and the analysis of only one time point, this study revealed new insights and pointed to a strong cell type-specific response to the presence of a conceptus^22^. Therefore, the aim of the present study was to perform a more extensive cell type-specific gene expression analysis by: (1) using an improved approach of the LCM and low-input RNA-sequencing that provide high quality RNA samples and RNA-seq libraries, and (2) analyzing different time points of pregnancy from day 10 to 13 to reveal new insights into MRP in the mare.

## Results

### Pregnancy rates, embryo sizes and histological evaluation of endometrial samples used for RNA-sequencing

Endometrial biopsy samples were collected on days 10, 11, 12, and 13 of pregnancy (P), and days 10 and 13 of the estrous cycle (C) from 11 mares of the originally 14 mares of the study, because three did not give an embryo in any of their P cycles. The 11 selected mares showed a pregnancy rate of 67%. For each of the 6 sampling days (pregnancy/cycle day: P10, P11, P12, P13 and C10, C13), 5 samples (biological replicates) were selected (total of 30 samples listed in Table S1). For days 10, 11, and 12, one out of the 5 pregnancies per group was a twin pregnancy (recovery of two embryos). For day 13, two of the 5 pregnancies were twin pregnancies. The recovered embryos had different sizes generally increasing with day, but also with high variation between embryos of the same day. The sizes of the embryos ranged from 3 to 5 mm, 3 to 10 mm, 4 to 11 mm, and 8 to 26 mm for days 10, 11, 12, and 13, respectively. Details are shown in Table S2. Histological sections of endometrial biopsy samples were evaluated and categorized according to Kenney and Doig into grade I, IIA, IIB or III^23^, depending on signs of inflammation and grade of endometrial fibrosis of the biopsies. The samples were graded between I and IIB, mostly I and IIA (Table S3).

### Tissue areas and RNA quality of the samples collected by LCM for RNA-sequencing

Luminal epithelium (LE), glandular epithelium (GE), and stromal areas (ST) were isolated from frozen sections of endometrial biopsies using LCM (Figs. S1 and S2). The average collected area for each endometrial compartment was 729,092 μm^2^, 689,755 μm^2^, and 4,155,773 μm^2^, respectively. The concentration of the isolated RNA ranged from 290 to 1626 (mean = 779.4) pg/μl, 304–1,386 (mean = 837.4) pg/μl, and 387–1,337 (mean = 736.4) pg/μl, respectively. The RNA integrity number (RIN) of these samples was 6.9–8.9, 7.1–8.8, and 6.2–8.1, respectively. A total of 90 low-input RNA-seq libraries were prepared and subjected to Illumina sequencing (Fig. S1).

### RNA-seq data shows a strong cell type-specific endometrial response towards embryo signaling

**Figure 1:**
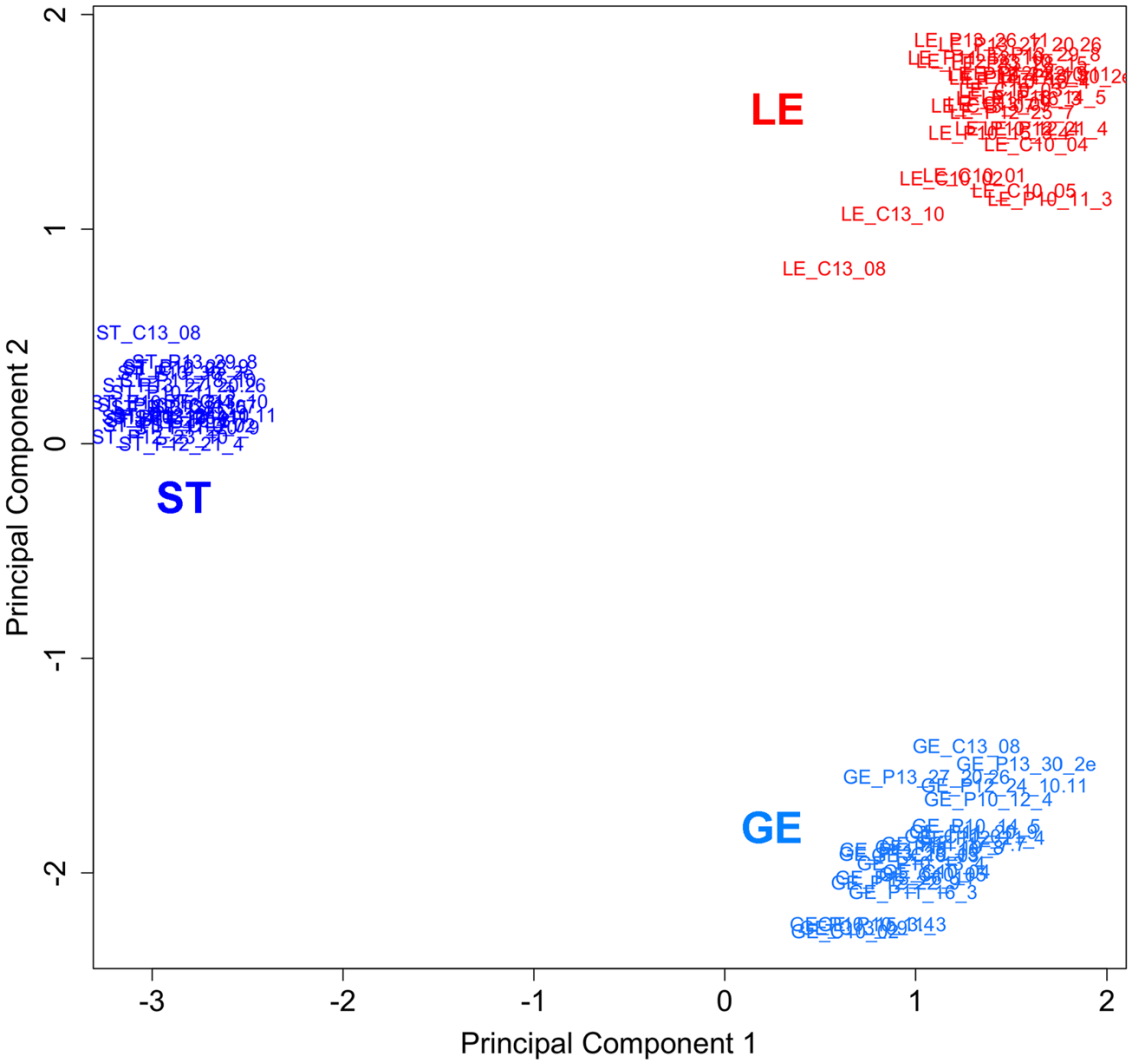
Clustering of endometrial luminal and glandular epithelium and stromal samples. Principal component analysis (EdgeR multi-dimensional scaling plot of the top 1000 genes with largest variation across all samples) showing a clustering of the endometrial samples depending on their cell type. *LE* luminal epithelium, *GE* glandular epithelium, *ST* stroma. Image created with Bioconductor package EdgeR (https://bioconductor.org/packages/edgeR/)^84^ and modified with Adobe Photoshop v.22.4.3.

First, a principal component analysis was performed with all samples from all cell types and days of C and P that passed the quality control (74 samples) (Fig. 1). A very clear grouping was found with a clear separation of stromal from the epithelial cells in the first dimension. In the second dimension, LE and GE were clearly separated. Analysis of DGE was performed for each cell type between P and C.

An initial analysis for each cell type between all P and C samples including all different days of P showed that P samples with small conceptuses (≤ 8 mm, mainly earlier stages of P) had expression profiles very similar to C samples, while P samples with larger conceptuses (≥ 8 mm, mainly later stages of P) showed quite distinct differences to the C samples (Fig. S3). Considering this finding, the main approach for analysis of DGE was based on two different comparative analyses: comparison 1; samples with larger conceptuses and clearly different gene expression profiles between P and C (LE: C n = 9, P n = 10; GE: C n = 7, P n = 10; stroma: C n = 6, P n = 12) and comparison 2; samples with smaller conceptuses and similar gene expression profiles between P and C (LE: C n = 9, P n = 7; GE: C n = 7, P n = 6; stroma: C n = 6, P n = 5).

**Figure 2:**
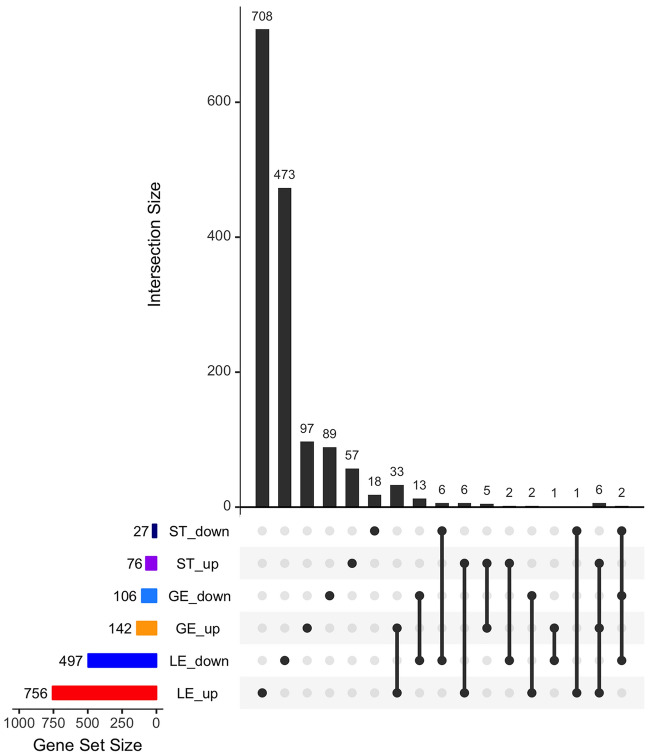
Results of differential gene expression analysis in comparison of samples with conceptus sizes ≥ 8 mm and cyclic controls (comparison 1). Overlap of differentially expressed genes between endometrial cell types illustrated by an Upset plot representing up- and downregulated genes obtained for luminal epithelium (LE), glandular epithelium (GE) and stroma (ST) (LE: FDR < 1%, p-value < 0.0009; GE: FDR < 5%, p-value < 0.0009; ST: FDR < 13%, p-value < 0.0009). Image created with the R package UpSetR v.1.4.0 (https://CRAN.R-project.org/package=UpSetR)^87^ and modified with Adobe Photoshop v.22.4.3.

The results of the first comparison for each cell type are shown in Fig. 2 and in the hierarchical cluster analysis in Fig. S4, revealing most differences between samples from P and C in LE with in total 1,253 differentially expressed genes (DEGs) (FDR < 1%, *p*-value < 0.0009), 756 upregulated and 497 downregulated in samples from pregnant mares. Less changes in gene expression were detected in GE and ST with 248 DEGs (142 up- and 106 downregulated; FDR < 5%, *p*-value < 0.0009) and 103 DEGs (76 up- and 27 downregulated; FDR < 13%, *p*-value < 0.0009), respectively (Fig. 2 and Fig. S4B,C). The overlap of the DEGs identified in the first comparison between cell types is also shown in Fig. 2. Only 6 upregulated and two downregulated genes were found in common for all three cell types. The relative overlap between GE and LE was slightly higher (18.5%) than for ST with LE (11.7%). In addition, a few genes were found with opposite regulation in comparison of LE with ST and LE with GE. The overlap analysis is pointing at a highly cell type-specific endometrial response to embryonic signals.

The second comparison also showed mainly differential expression in LE with in total 248 DEGs (FDR 5%, *p*-value < 0.001), 47 with lower and 201 with higher expression in P samples compared to C samples. At the same FDR (5%), only 43 DEGs were obtained for GE (31 lower and 12 higher in P) and 64 DEGs for ST (32 lower and 32 higher in P). Differential expression analysis was also performed for LE between cyclic samples of days 10 and 13 since for this cell type 5 and 4 biological replicates, respectively, were available per day after quality filtering. This comparison revealed 377 DEGs (FDR 5%), 237 with higher expression on day 10 and 140 with higher expression on day 13 of the estrous cycle. All details for detectable genes in LE, GE, and ST are shown in Tables S4–S6, respectively.

### Clustering of LE samples according to their expression profiles

**Figure 3:**
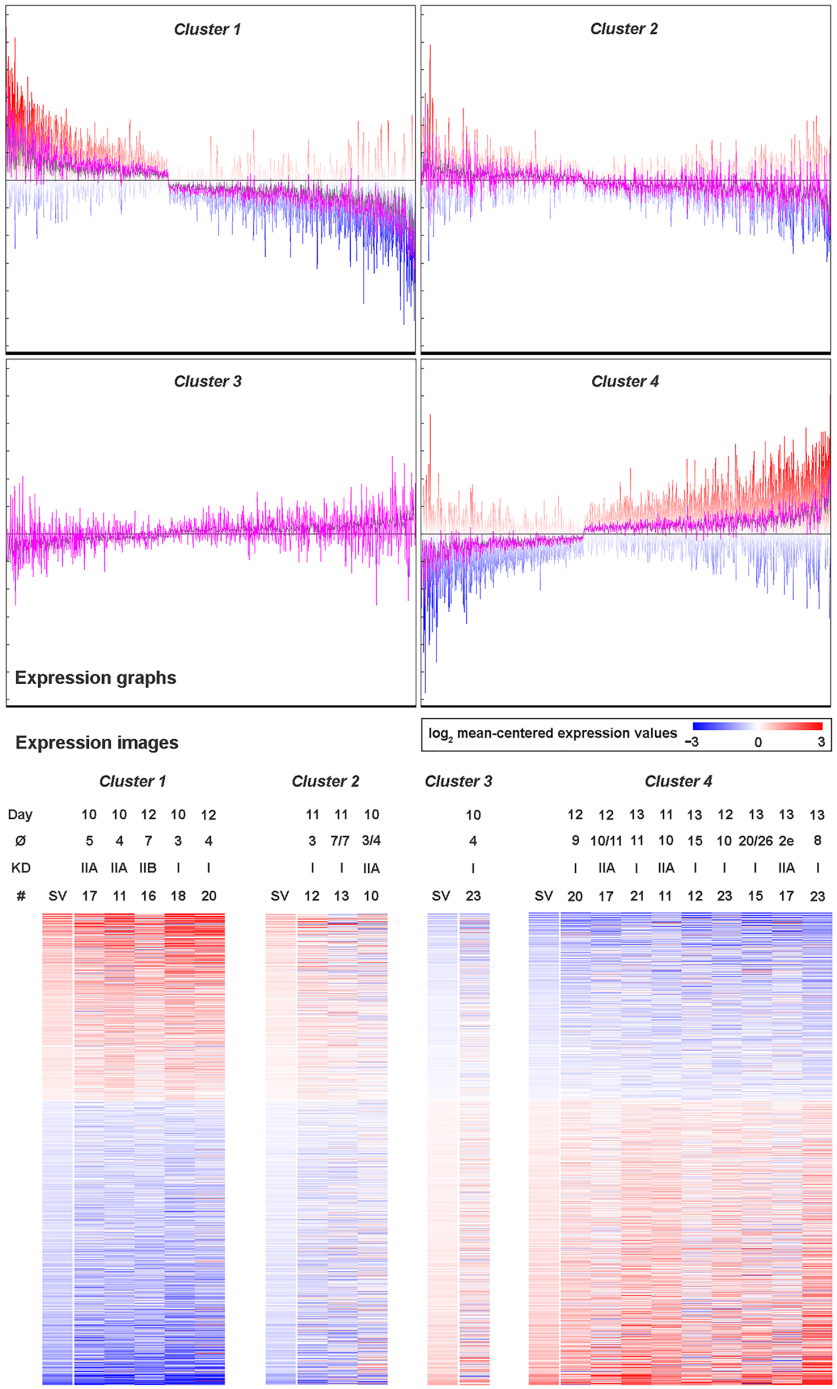
Clustering of luminal epithelium (LE) samples derived from day 10–13 pregnant mares. Self-organizing Map (SOM) analysis of all LE samples was performed based on the differentially expressed genes identified in comparison 1 resulting in 4 clusters of samples collected from day 10–13 pregnant mares. Expression graphs of the clusters are shown in the upper part being cluster 1 the most similar to cyclic controls (see Fig. S5) and cluster 4 the most different. The lower part shows the expression images. Day of pregnancy, size of conceptus(es), Kenney and Doig score, and number of the mare are shown. The color scale illustrates the log2-mean centered expression values (log2 counts per million of the sample minus mean of all samples) from blue (− 3) over white (0) to red (+ 3). Image created with Multiple Experiment Viewer (MeV v.4.8.1, https://sourceforge.net/projects/mev-tm4/)^88^ and modified with Adobe Photoshop v.22.4.3.

A self-organizing maps (SOM) analysis was performed to cluster all 19 LE P samples according to their expression profiles of the 1,253 DEGs obtained in comparison 1. The result of this analysis revealed grouping into 4 different clusters (Fig. 3). Figure 3 also shows conceptus sizes, twin pregnancies, and Kenney and Doig scores. Most of the samples assembled in cluster 1 or 4. Cluster #1 (n = 5) mainly contained samples where smaller embryos were found (size range of 3–7 mm in diameter) collected on P days 10 and 12. Cluster #2 included 3 samples from P days 10 and 11 and different embryo sizes (3–7 mm) including two twin pregnancies. Cluster #3 only consisted of one sample collected on day 10 of P with an embryo of 4 mm in diameter. The samples of cluster #4 (n = 9) were mainly derived from pregnancies of later stages (except one day 11 sample, all from days 12 and 13) with embryos ranging from 8 to 26 mm in diameter. The expression profiles in cluster #1 where the most similar compared to the C sample profiles, whereas cluster #4 contained the samples with the most different expression profiles in comparison to the C group (see Fig. S5).

### Functional annotation analysis and pathways of comparison 1 DEGs of LE, GE and ST

The most significantly enriched functional terms (clusters of related terms) for genes upregulated in LE are shown in Table 1. The overrepresented functional categories belonged to biological processes involved in secretion and vesicle transport, signal transduction, cytoskeleton, cell and focal adhesion, blood vessel development, and biosynthetic and phospholipid metabolic processes. A lower number of significantly overrepresented functional annotation clusters was obtained for downregulated genes in LE. These clusters were mainly associated with lipid, steroid/sterol, and amino acid metabolic processes, morphogenesis and development, and response to growth factors (Table 2). Table S7 shows all results obtained by Metascape analysis.

**Table 1:** Selected functional enrichment results for genes upregulated in luminal epithelium of pregnant mares (comparison 1) identified by MetaScape.

Summary ID	Category	Term ID	Description	Log (q-value)	DEGs/total genes in term
1	CC	GO:0016324	Apical plasma membrane	− 8.71	38/363
2	BP	GO:0007169	Transmembrane receptor protein tyrosine kinase signaling pathway	− 7.09	53/753
3	CC	GO:0070161	Anchoring junction (focal adhesion)*	− 6.80	44/570
4	BP	GO:0009611	Response to wounding (coagulation)*	− 6.80	50/708
5	CC	GO:0030659	Cytoplasmic vesicle membrane (leukocyte degranulation)*	− 6.80	53/784
6	BP	GO:0001568	Blood vessel development	− 6.66	53/794
7	BP	GO:0033674	Positive regulation of kinase activity (MAPK cascade)*	− 6.56	45/615
8	BP	GO:0030036	Actin cytoskeleton organization	− 6.56	49/708
9	BP	GO:1902532	Negative regulation of intracellular signal transduction	− 6.34	43/581
10	BP	GO:1901652	Response to peptide (response to insulin)*	− 6.28	41/540
11	BP	GO:0097190	Apoptotic signaling pathway	− 6.23	44/612
12	BP	GO:0051345	Positive regulation of hydrolase activity (regulation of GTPase activity)*	− 6.20	51/783
13	CC	GO:0010008	Endosome membrane	− 5.79	38/501
15	BP	GO:0030155	Regulation of cell adhesion (lymphocyte activation)*	− 5.79	48/738
17	MF	GO:0031267	Small GTPase binding (Ras/Rab GTPase binding)*	− 5.03	33/433
20	KP	ko04144	Endocytosis	− 4.73	24/260
22	BP	GO:0071396	Cellular response to lipid (response to glucocorticoid, steroid hormone)*	− 4.41	38/590
23	BP	GO:0071363	Cellular response to growth factor stimulus (response to TGFB, transmembrane receptor protein Ser/Thr kinase signaling pathway)*	− 4.36	43/719
25	BP	GO:0034330	Cell junction organization (cell–cell junction, tight junction assembly)*	− 4.34	26/318
30	BP	GO:0008610	Lipid biosynthetic process (phospholipid, phosphatidylinositol metabolic process)*	− 4.21	43/733
33	BP	GO:0030336	Negative regulation of cell migration	− 4.16	27/350
39	BP	GO:0001890	Placenta development (in utero embryonic development)*	− 3.97	17/157

*CC* cellular components, *BP* biological processes, *MF* molecular functions, *KP* KEGG pathways.

*Additional terms added from member categories.

**Table 2:** Selected MetaScape functional enrichment results for genes downregulated in luminal epithelium of pregnant mares (comparison 1).

Summary ID	Category	Term ID	Description	Log (q-value)	DEGs/total genes in term
1	BP	GO:0006767	Vitamin metabolic process (cellular modified amino acid metabolic process)*	− 2.94	11/89
2	BP	GO:0048736	Appendage development (embryonic morphogenesis)*	− 2.94	15/181
4	BP	GO:0030036	Actin cytoskeleton organization (actin binding)*	− 2.67	30/708
5	MF	GO:0042578	Phosphoric ester hydrolase activity (dephosphorylation)	− 2.30	20/376
6	BP	GO:1901615	Organic hydroxy compound metabolic process (steroid, sterol metabolic process)*	− 2.30	25/556
7	BP	GO:0070848	Response to growth factor (response to TGFB)*	− 2.30	30/749
8	BP	GO:0048858	Cell projection morphogenesis (chemotaxis)*	− 2.23	28/685
9	BP	GO:0006323	DNA packaging (chromatin remodeling, nucleosome organization)*	− 2.14	14/210
10	BP	GO:0032787	Monocarboxylic acid metabolic process (fatty acid metabolic process)*	− 2.14	27/664
11	CC	GO:0031012	Extracellular matrix (matrisome-associated)*	− 2.08	24/569
12	BP	GO:0002064	Epithelial cell development	− 2.01	14/226
13	BP	GO:0048729	Tissue morphogenesis (epithelial to mesenchymal transition)*	− 1.89	26/668
16	BP	GO:0045596	Negative regulation of cell differentiation (regulation of cell projection organization)*	− 1.73	28/772
18	BP	GO:0008610	Lipid biosynthetic process (phosphatidylinositol, glycerolipid metabolic process)*	− 1.44	26/733

*CC* cellular components, *BP* biological processes, *MF* molecular functions.

*Additional terms added from member categories.

The genes identified as differentially expressed (DE) in GE were enriched for terms related to response to steroid and peptide hormones, extracellular matrix, carbohydrate metabolism, focal adhesion, blood vessel development, and negative regulation of immune system processes (Table S7). The functional terms overrepresented for the DEGs in ST were associated with leukocyte activation, transmembrane receptor protein tyrosine kinase signaling pathway, and lipid transport (Table S7).

A comparison of all overrepresented functional terms, biological processes, and canonical pathways obtained for the DEGs identified in LE, GE and ST by using Metascape and Ingenuity Pathway Analysis (IPA) software is shown in Fig. S6. Terms and pathways specifically enriched for LE were for example related to various signaling pathways, lipid metabolism, vesicle-mediated transport, and cytoskeleton organization (Fig. S6A,B). Canonical pathways specifically enriched for LE and with a positive activation Z-score (activated according to the regulation of the corresponding DEGs) were, e.g., IL-15 production, neuregulin signaling, ErbB signaling, signaling by Rho family GTPases, Wnt/β-catenin signaling, and TGF-β signaling (Fig. S6C). Furthermore, several canonical pathways with a negative activation Z-score were found, e.g., cholesterol biosynthesis, RhoGDI signaling, and PPARα/RXRα activation (Fig. S6C). Functional terms and pathways specifically overrepresented for GE and ST were related to NABA core matrisome, hexose metabolic process, apoptosis, activation of blood cells, and immune response signaling (Fig. S6).

### Upstream regulator analysis for each cell type

Potential upstream regulators of the DEGs identified in comparison 1 were obtained with Metascape^24^ and IPA software (Fig. S7). The IPA upstream regulator analysis revealed several factors as significantly overrepresented and with a positive Z-score for LE, such as CREB1, TNF, interleukin 1 beta (IL1B), epidermal growth factor (EGF), TGFB1, leukotriene D4, ERK, VEGF, RAF1, beta-estradiol (E2), and MAPK3 (Fig. S7A). In LE, *EGF* and *IL1B* mRNAs were found as upregulated (log2 fold change 4.6 and 1.0). Interleukin 1 beta (IL1B) was also found as significant and with a positive Z-score for GE. In contrast, TGFB1 had a negative Z-score for GE. Specific overrepresentation and activation for GE was found for FOXO3 and KRAS. Progesterone was specifically overrepresented and activated for stroma. Metascape analysis revealed genes regulated by estrogen receptor 1 (ESR1) only as overrepresented for LE (Fig. S7B). In addition, genes that are regulated by Sp1 transcription factor (SP1) and RELA proto-oncogene, NF-kB subunit (RELA) were enriched for LE, GE, and ST. Genes regulated by peroxisome proliferator activated receptor alpha (PPARA), transcription factor AP-2 alpha (TFAP2A), and SP3 were enriched for LE and GE.

**Figure 4:**
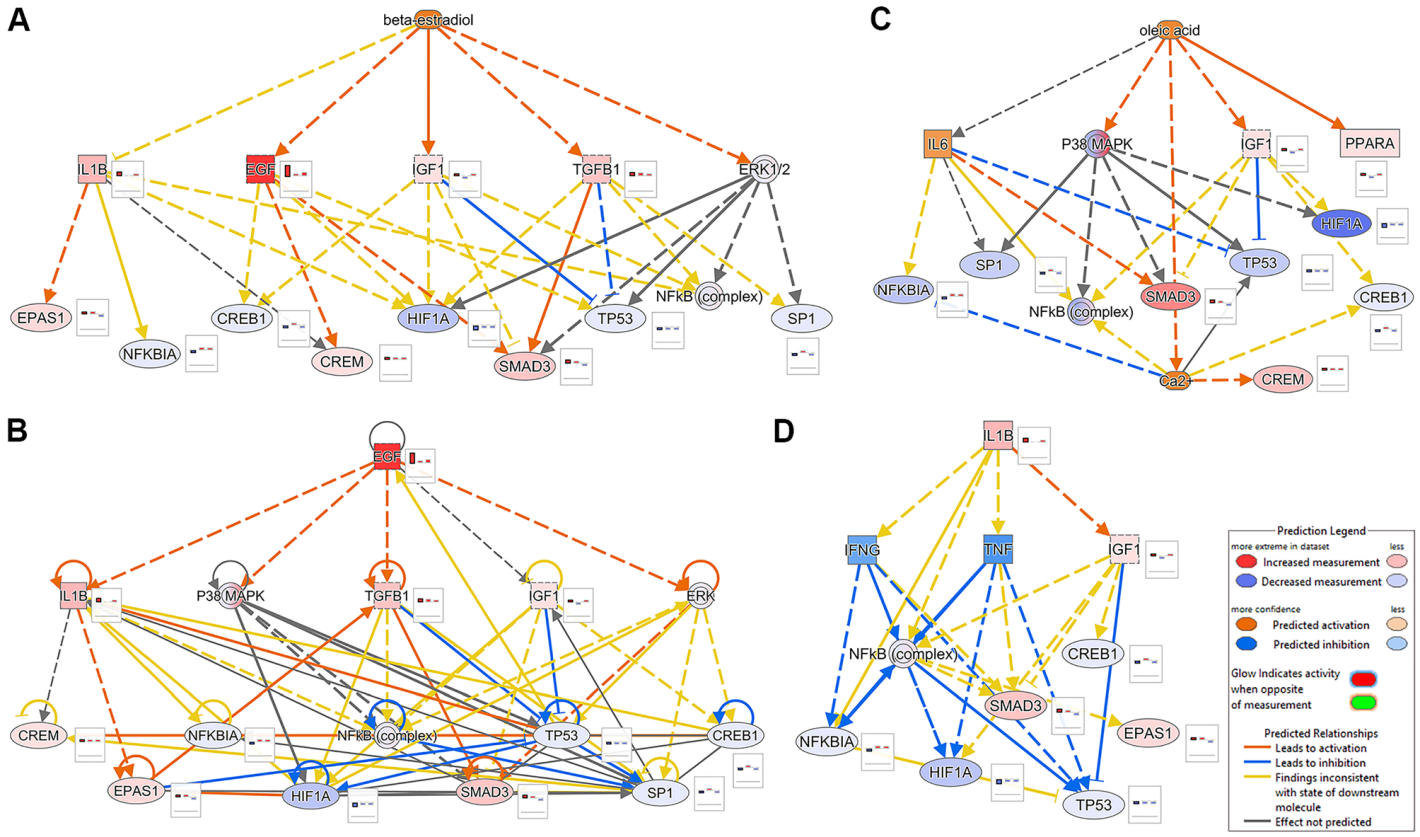
Upstream regulator networks of potential upstream regulators of the DEGs identified for luminal epithelium (LE) in comparison 1. Potential upstream regulators were identified using Ingenuity Pathway Analysis software. Regulator networks are shown for (**A**) beta-estradiol, (**B**) epidermal growth factor (EGF), (**C**) oleic acid, and (**D**) interleukin 1 beta (IL1B). For genes detected as expressed in LE, log2 fold change is shown in the bar plot next to the gene symbol for LE, glandular epithelium, and stroma (from left to right). Image created with Ingenuity Pathway Analysis software v.68752261 (https://digitalinsights.qiagen.com/products-overview/discovery-insights-portfolio/analysis-and-visualization/qiagen-ipa)^86^ and modified with Adobe Photoshop v.22.4.3.

The overlap of the target genes of the obtained upstream regulator networks for the DEGs found in LE was analyzed by using Venn diagrams (Figs. S8 and S9). The analysis of these overlaps suggested, e.g., the potential upstream regulators E2, oleic acid, EGF, and IL1B and connected downstream regulators as causal for DGE in LE. These regulator networks are shown in Fig. 4A–D.

### Overlap and comparative functional annotation analysis of DEGs in luminal epithelium of comparisons 1 and 2

For LE, the overlap of DEGs between comparison 1 and 2 and the comparison between C10 and C13 was analyzed. Almost half of the DEGs obtained in comparison 2 (smaller conceptuses vs. cyclic controls) were also found in comparison 1 (Table S8). Comparative functional annotation and upstream regulator analysis (Fig. S10) revealed a high similarity of overrepresented functional terms and pathways (Fig. S10A-C) and potential upstream regulators (Fig. S10D,E). A few categories and pathways, and potential regulators were more significant for comparison 2, such as TF-related pathways, regulation of fatty acid (FA) oxidation, xenobiotic metabolism CAR (NR1I3) signaling, adrenomedullin signaling, estrogen receptor signaling, insuline like growth factor 1 (IGF1), and interleukin 6 (IL6) (Fig. S10A–E). Strikingly, several TF described as “early response genes” of the TF complex activator protein 1 (AP-1), early growth response (EGR), Kruppel like factor (KLF), and nuclear receptor families were already upregulated in LE in comparison 2.

### Membership analysis to identify genes involved in prostaglandin signaling and metabolism, and mechanical sensation

Using the webtool Metascape, a membership analysis was performed for the DEGs identified in LE (comparisons 1 and 2) with the keywords “prostaglandin” (PG), “arachidonic acid” (AA), and “mechanical”. Table S9 shows the identified genes (in total 76), their regulation in LE, and assignment to various functional annotation terms. For PG, 33 genes were assigned, 12 for AA, and 37 for “mechanical”. Some genes were found as associated to more than one keyword. Two of the genes known as directly involved in PG metabolism, 15-hydroxyprostaglandin dehydrogenase (*HPGD*) and prostaglandin reductase 1 (*PTGR1*), were already found as upregulated in comparison 2.

### Searching for specific gene expression profiles in LE across cycle and pregnancy samples indicating potential association with MRP and establishment of pregnancy

**Figure 5:**
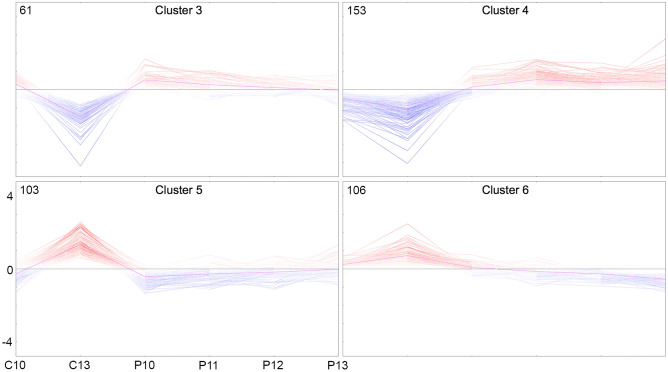
Identification of genes up- or downregulated in luminal epithelium (LE) from day 10 to day 13 of the estrous cycle but unchanged in pregnancy. Mean log2 counts per million (cpm) values were calculated for each day of C and P for genes DE in LE between pregnant and cyclic samples (FDR 1%) and between day 13 and day 10 of C (FDR 5%). The log2 cpm values per day were used to calculate mean-centered expression values (log2 cpm of the sample minus mean of all samples). Self-organizing tree algorithm analysis was used to identify clusters of genes with similar expression profiles. Four of the in total 9 clusters (see Fig. S12) are shown here. The number in the top left corner of each graph represents the number of genes in the cluster. C10/C13: days 10/13 of cycle; P10–P13: days 10 to 13 of pregnancy. Vertical axis: mean-centered expression values in log2 scale. Image created with Multiple Experiment Viewer (MeV v.4.8.1, https://sourceforge.net/projects/mev-tm4/)^88^ and modified with Adobe Photoshop v.22.4.3.

Genes found as differentially expressed in LE between days 10 and 13 of C and between P and C in comparison 1 (Table S4) were analyzed by self-organizing tree algorithm (SOTA, Multi Experiment Viewer software^25^). This analysis revealed 9 clusters of genes with similar expression profiles over days 10 and 13 of C and days 10, 11, 12, and 13 of P (Fig. S11). Four of these profiles were particularly interesting since they displayed: (i) genes downregulated from day 10 to day 13 of C but not from day 10 to day 13 of P (Fig. 5, clusters 3 and 4); and (ii) genes upregulated from day 10 to day 13 of C but not from day 10 to day 13 of P (Fig. 5, clusters 5 and 6). Particularly, cluster 4 contained genes found already as upregulated in comparison 2.

The DEGs in LE with the specific profiles shown in Fig. 5 were further filtered based on expression differences between days 13 of P and day 13 of C (log2 FC ≥ 1) to obtain genes which expression was either decreasing from day 10 to day 13 of C but unchanged or upregulated in P samples (“P13 up”) or increasing from day 10 to day 13 of C but unchanged or downregulated in P samples (“P13 down”) (Table S10).

**Figure 6:**
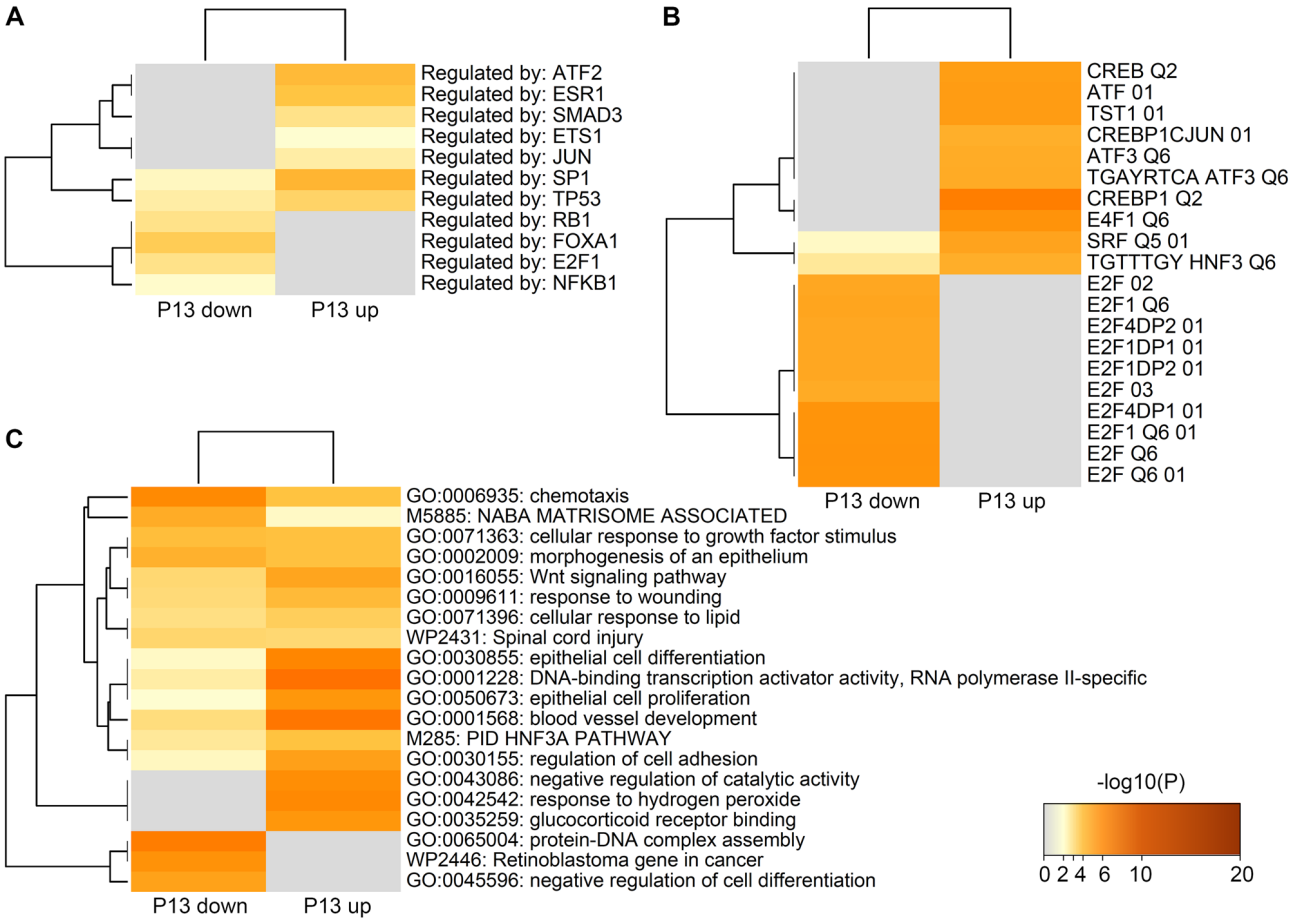
Overrepresented regulators, transcription factor binding sites, and functional categories for differentially expressed genes (DEGs) in luminal epithelium (LE) between cycle day 13 and 10. Genes of the clusters shown in Fig. 4 were selected based on the expression differences between days 13 of P and day 13 of C (log2 FC ≥ 1) to obtain genes which expression was either decreasing from day 10 to day 13 of C but unchanged in P samples (clusters 3 and 4, “P13 up”) or increasing from day 10 to day 13 of C but unchanged in P samples (clusters 5 and 6, “P13 down”). Metascape analysis was performed for the corresponding gene lists. (**A**) Potential upstream regulators (TRRUST database). (**B**) Overrepresented transcription factor binding sites (Transcription Factor Targets database). (**C**) Overrepresented functional terms, biological processes, and canonical pathways. The heatmaps are colored by statistical significance (– log10 of P-value) from gray (not significant) to brown (highly significant). Image created with Metascape webtool (https://metascape.org)^24^ and modified with Adobe Photoshop v.22.4.3.

Upstream regulator analysis for these two groups of genes revealed basically the same factors as identified for the DEGs in LE in comparison 1 (Fig. 4). Results of Metascape analysis are shown in Fig. 6. Overrepresentation analysis for transcription factors revealed FOXA1, E2F1 and MYCN (Fig. 6A,B P13 down) as potential regulators of the genes upregulated on day 13 of C (Fig. 5, clusters 5 and 6) and SP1, ATF2-4, TST1 (POU3F1), SRF, and ESR1 (Fig. 6A,B P13 up) as potential regulators for the genes downregulated on day 13 of C (Fig. 5, clusters 3 and 4). Furthermore, the overrepresentation analysis of functional categories showed specific functional terms for the two groups of genes (Fig. 6C and Fig. S12).

The genes assigned to “P13 up” and “P13 down” contained a strikingly large number of transcription factors (TF), e.g., members of the SRY-box TF family, KLF family, and nuclear receptor family (NR4A and NR2F) among others (Table S10). The latter family contained also nuclear receptor subfamily 2 group F member 2 (*NR2F2,* found as upregulated in LE comparison 1 and 2) which was found to interact with many other DEGs in LE, based on IPA interaction network analysis (Fig. S13). *NR2F2* expression levels were low in GE at similar levels compared to LE of cyclic samples and high in stroma in both cyclic and pregnant samples (Tables S4–S6). Expression of many of the above-mentioned TF was already upregulated in LE in comparison 2 (Table S4 comparison 2 and Table S10).

## Discussion

Our study provides the first extensive spatiotemporal transcriptome study in the mare covering different time points during the window of MRP. The approach used in our study to unravel unknown MRP signaling in the mare was based mainly on three points: (i) up to date, it is not completely clear when MRP is occurring in the mare; (ii) it is expected that the endometrial response to conceptus signaling depends on the developmental stage of the conceptus; and (iii) previous studies on cyclic control samples collected from day 10 to day 13 showed quite similar gene expression on these days of the estrous cycle^26^. Therefore, endometrial samples were collected on days 10, 11, 12, and 13 of P (days after ovulation) from 5 mares per P day and only on days 10 and 13 from cyclic mares. Moreover, effort was directed to collect a good number of endometrial samples from these pregnancy days that could contain embryos with different sizes.

Overall, the average sizes of the recovered conceptuses gradually increased from day 10 to day 13 after ovulation, but with expected variability for the same day of P as it has been described for this period of rapid growth^27^. Whilst most of the conceptuses were in the normal range, one collected on day 12 was only 4 mm in diameter, suggesting arrested development. The data of this sample was not used for DGE analysis. On the basis of the experiences in our previous studies^11^ and the results of others^12,14^, a robust approach for identification of DGE was used based on a higher number of biological replicates. Particularly, the studies of Klohonatz et al.^12,14^ indicated that a low number of replicates can lead to controversial results since they did not find DEGs on day 12 of P in their first study^28^, but later they even identified DEGs on days 9 and 11 of P^12^. Unfortunately, these and other authors did not provide information about sizes of obtained conceptuses. A first analysis considered all samples collected and showed that the conceptus size could have a stronger effect than the day of P. Thus, the further approach was based on performing two different comparative analyses by grouping samples with larger conceptuses (≥ 8 mm, comparison 1) and samples with smaller conceptuses (≤ 8 mm, comparison 2) and comparing both to the same controls (C10 and C13). This approach agrees with the assumption that larger conceptuses will induce a stronger endometrial response and revealing a higher number of DEGs in comparison 1 and a much lower number in comparison 2. This contrasts with Klohonatz et al.^12^, where they found the same number of DEGs on days 9 and 11 and less on day 13. However, this result was probably due to the very low number of replicates (n = 3).

One of the main findings in the present study was that (i) the main response occurred in the endometrial LE and (ii) the response is highly specific with very low overlap between LE, GE, and ST, which confirmed results of a pilot study from our group on day 12 of pregnancy^22^. Similar results were obtained for porcine endometrium on days 12 and 14 of pregnancy^20,21^. Specific gene expression in endometrial LE and GE has been reported also in human^29^ and mouse^30^. The localization of the main response to the LE is in agreement with the known signaling molecules secreted by the equine conceptus and the continues migration throughout the uterus which are mainly acting on the uterine luminal surface^2^.

The much lower number of DEGs observed in comparison 2 (small conceptuses) is also in agreement with our previous study^11^, where no DEGs could be detected on day 8 of pregnancy compared to day 8 of the estrous cycle. In our previous studies^18,19^, we also compared DGE between days 12 and 16 of P revealing a great overlap of DEGs and just more pronounced differences on day 16 compared to the day 12 cyclic controls. Since the study of day 8 of P^11^ was not performed with dissected endometrial samples, it could be possible that DGE occurs in LE already on day 8 or 9, i.e., when a conceptus < 3 mm is present. Further studies on this earlier window with the LCM-RNA-sequencing approach could show if there is an earlier response than so far expected.

The following paragraphs are discussing the results of data mining with respect to the potential association of the identified DEGs with MRP and establishment of pregnancy. Because the observed gene expression changes were on the transcript level, the interpretations have some speculative character and are rather intended to provide starting points for in depth functional studies.

Overall, functional term overrepresentation analysis suggested several molecular pathways as involved in embryo sensing and establishment of pregnancy. Presumed effects of E2 and other factors known to be secreted by the conceptus and the effects of the close mechanical contact to the conceptus capsule, were reflected in corresponding overrepresented functional terms for genes up- or downregulated in LE, e.g., enrichment of signaling pathways and response to growth factors in the genes upregulated in LE of pregnant mares. Several members of a novel signaling pathway found in LE cells in sheep^31^, named JAK-SRC kinase-EGFR-RAS-RAF-ERK1/2-early growth response (EGR)-1 signaling module, were upregulated in LE of pregnant mares (e.g., *EGF*, *MAP2K1*, *MAPK13*, *MAP3K21*, *EGR1-3*), most of them in both comparison 1 and 2. This signaling pathway is involved in prevention of pulsatile release of PGF2a via regulation of PG transporter protein in ovine endometrium^31^. Regarding further regulatory pathways involved in MRP in other species, *IRF2* mRNA was found as specifically upregulated in LE similar to ovine^32^ and porcine endometrial LE on day 12 of pregnancy^33^ and upregulation of *ESR1* mRNA was observed on day 13 of the estrous cycle but not day 13 of P (log2 FC = – 1.6) in agreement with data from day 13.5^15^. Together these findings suggest similar signaling pathways but leaving the question which signaling molecule is inducing these responses in the LE. As in other mammals, oxytocin receptor has been shown to mediate the pulsatile release of PGF2a in the mare after induction by oxytocin^34^. In contrast to pigs and ruminants, *OXTR* mRNA was upregulated in LE of pregnant mares, in agreement with a previous study showing downregulation of OXTR at the protein level^35^. However, OXTR expression increased between days 14 and 21 of P suggesting that OXTR expression increase is only delayed and other components are involved in preventing luteolytic pathway generation^35^. De Ruijter-Villani et al. suggested that downregulation of prostaglandin F receptor (*PTGFR*), which they found on days 14 and 21, as a mechanism to uncouple the oxytocin-PGF2α feedback loop^35^. Here, we found only very low expression of *PTGFR* mRNA regardless day or stage.

The overrepresentation of cell adhesion, cell junction, and cytoskeleton organization for genes upregulated in LE could be related to the plasma membrane transformation in LE of human endometrium that facilitates endometrial receptivity and implantation^36^. The plasma membrane transformation shows parallels to the epithelial-mesenchymal transition and includes morphological and molecular alterations involving the actin cytoskeleton, remodeling of adherence and tight junctions, mucins, integrin expression, and epithelial-stromal communication. In equine LE, various genes known to be involved in this process were found as differentially expressed, e.g., genes coding for components and regulators of the actin cytoskeleton, adherence, tight and anchoring junctions, beta integrins, and mucins. In contrast to humans, several genes known as involved in human embryo implantation such as *WNT5A*, *ITGA2*, and mucin genes *MUC4* and *MUC2*^37^ showed opposite regulation or were unchanged in LE, suggesting a role in regulation of delayed implantation in the mare. Interestingly, the mRNAs of claudins 4 and 10 (*CLDN4*, *CLDN10*) show a conserved pattern of increased endometrial expression during the receptive phase in cattle, pig, horse, and human^18,38^.

In addition to PG metabolism and signaling (discussed below), genes up- or downregulated in LE related to FA and lipid metabolic processes were overrepresented. Many of these genes were involved in inositol phosphate and sphingolipid metabolism and signaling. Upregulation in endometrial LE of genes participating in sphingolipid metabolism, e.g., ceramide synthase 4 (*CERS4*), sphingomyelin phosphodiesterase 1 (*SMPD1*), and sphingomyelin synthase 1 and 2 (*SGMS1/2*) could have regulatory roles at the maternal-embryonic interface as shown in the mouse^39^. Mizugishi et al.^40^ found in mice and humans that sphingolipid metabolic pathway regulates innate immunity at the fetomaternal interface and thereby plays a critical role in fetomaternal tolerance. Genes, involved in FA metabolism and biosynthesis of unsaturated fatty acids (UFA) could be connected to the synthesis of arachidonic acid (AA), the precursor of PGs. In porcine endometrium, downregulation of mRNA expression of genes involved in generation of AA precursors was found suggesting this as a mechanism to decrease PG production^21,41^. In contrast, many of these genes were upregulated in equine endometrial LE, which could point to a role in provisioning the conceptus with lipids for metabolism as well as morphogenesis and pattern formation^42^.

Due to their known importance for MRP, a focus of the data analysis was on genes involved in PG signaling and metabolism. In addition to the above-mentioned upregulation of OXTR mRNA, *PTGER2* mRNA was found with lower levels in comparison 1. Atli et al.^43^ found upregulation of *PTGER2* during late diestrus (days 13.5–14 post-ovulation), early luteolysis, and pregnancy days 14–22 and a downregulation of the receptor after luteolysis. Gebhardt et al.^26^ analyzed different days of the cycle and found decreased levels of *PTGER2* mRNA on day 16 and increased levels on days 8 and 12 post-ovulation. While diverse functions of PTGER2 in endometrium of different mammals have been suggested, a recent study showed induction of prostaglandin endoperoxide synthase 2 (*PTGS2*) and growth factor gene expression after PTGER2 activation in bovine endometrial epithelial cells^44^.

Phospholipase A2 (PLA2) family members have been suggested to be involved in regulation of endometrial PGF2a synthesis in the mare and DGE of PLA2 isoforms has been shown during estrous cycle and early pregnancy^45,46^. In the present study, phospholipase A2 group IIA (*PLA2G2A*) mRNA was decreased in comparison 1 and *PLA2G4A* mRNA was decreased in LE on day 13 in comparison of pregnant and cyclic mares, further supporting a role in controlling PGF2a synthesis and luteolysis.

Another way to regulate PG effects is via PG transporters to control local PG concentration. The increased mRNA expression of *SLCO2A1* in comparison 1 in LE indicated a role in regulation of PG transport, particularly of PGE2 and PGF2a in LE. In the pig, this gene was also found as upregulated in LE^33^. Our previous studies showed differential expression of *SLCO2A1* mRNA during the estrous cycle with lowest expression on days 3 and 8 and highest on day 16^26^. In contrast, Atli et al.^43^ and Ruijter-Villani et al.^35^ did not detect differences during the period of MRP and the estrous cycle, respectively. However, these authors did not perform cell type-specific expression analysis and did not analyze the same days of C. In pigs, *SLCO2A1* is mainly expressed in endometrial LE and blood vessels^21,47^, and an important role in regulation of PG transport at the fetomaternal site and establishment of pregnancy has been suggested^47,48^. In humans, upregulation of *SLCO2A1* expression has been found in decidual stromal cells^49^, and the authors showed that PG uptake (mainly PGE2) by decidual cells is mediated by SLCO2A1. Thus, in the mare SLCO2A1 could have a similar function in LE. Another PG transporter, ATP binding cassette subfamily C member 1 (*ABCC1*), was also found as upregulated in comparison 1 in LE. In the same line, *ABCC1* expression was mainly localized in LE and GE and was increased by E2 in pregnant gilts^48^.

In addition to synthesis and transport of PG, degradation is another option for PG regulation. The observed regulation of two genes involved in PG degradation (*PTGR1* upregulated in LE in comparison 1 and 2; *HPGD* only in comparison 2) suggests this option as a way to prevent PGF2a release from the endometrium in the mare. Both genes are involved in degradation of PGE2 and other PGs where PTGR1 is catabolizing 15-keto-metabolites resulting from initial oxidation catalyzed by HPGD into the fully inactive 13,14-dihydro-15-keto-metabolites^50^. Prostaglandin reductase 1 also catalyzes the oxidation of leukotriene B4 (LTB4) to inactive12-keto-LTB4^50^. Altogether, the DGE identified in the LE indicated regulation of PGE2 receptors, PG precursor generation, PG transport, and PG metabolism as potential processes involved in regulating endometrial PGF2a release into the circulation.

The results of the regulator network analysis indicated complex effects and suggested in addition to E2, cytokines (e.g., IL1B, TNF or IFNG) or substances with similar effects as well as FAs or other lipids as potential regulators of the embryo signaling response. The fact that some classical downstream regulators of E2 and other factors were not found as upregulated or even downregulated or not detectable at mRNA level, suggested that several signaling molecules are involved affecting each other’s downstream regulatory network. For example, *ERRFI1,* strongly upregulated in LE in this and in a previous study^16^, has been described as critical for implantation in mice by suppressing ESR1 activity in the uterine epithelium and mediating P4′s suppression of E2 signaling during embryo implantation^51^. Furthermore, *ERRFI1* is acting as a negative feedback regulator of EGF receptor signaling and is stimulated by epidermal growth factor^52^ which mRNA was highly upregulated in LE in P.

To identify specific candidate genes involved in regulation of MRP in the mare, a focus of the data analysis was on genes which showed already DGE between P samples with smaller conceptuses (comparison 2) and on genes with specific expression profiles across the analyzed days of C and P. Genes with early up- or downregulation and genes with increasing or decreasing expression on day 13 of C while unchanged for P days could be involved in establishment of pregnancy and possibly in MRP.

Particularly the genes upregulated in P in comparison 2 were highly overrepresented for TF. Many of those belonged to the so-called early response TF such as the AP-1 complex subunits (FOS, FOSB, JUN, JUNB), KLF members, EGR1-3, and nuclear receptors. This could be another indication that signals of conceptuses ≤ 3 mm in diameter are inducing the first endometrial response since these factors are known to be rapidly and transiently induced after signaling by various external stimuli^53^.

Four members of the KLF TF family (*KLF2*, *KLF4*, *KLF6*, *KLF9*) showed a similar expression profile in LE with decreasing levels on day 13 of C but increased levels on day 13 of P. *KLF6* and *KLF9* showed already higher expression in LE of P samples in comparison 2. The expression profiles and high overrepresentation of KLF TF are suggesting an important role in the LE in establishment of pregnancy. Since KLF6 has been shown to inhibit ESR1-mediated cell growth in breast cancer cells^54^, it could be involved in suppression of estrogen effects on cell growth in equine endometrium. Zhang et al.^55^ showed that KLF9 and PGR are coregulatory proteins mediating P4 responsiveness of target genes in endometrial cells. A KLF9 knockout in the mouse resulted in subfertility, uterine hypoplasia, and partial P4 resistance showing the general importance of *KLF9* expression in the endometrium during pregnancy^56^. In human endometrium, PGR agonists induced *KLF4* expression leading to inhibition of cell proliferation, suggesting KLF4 as a mediator of anti-proliferative effects of P4^57^. The family members KLF2 and KLF4 are belonging to the group of mechanosensitive TF^58^, pointing at a possible regulation by the mechanical stimulus of the migrating conceptus.

Furthermore, DGE of several members of the SRY-box TF family were identified in LE. The most interesting seemed to be *SOX17*, which has been found as upregulated in human endometrial epithelial cells by combined estrogen and P4 during the receptive window^59^. Knockdown or inhibition of SOX17 prevented trophoblast cell attachment^59^, and a decrease in the number of implantation sites was found in Sox17 heterozygous mutant mice^60^. Although, the actual conceptus implantation in the horse is happening much later, *SOX17* expression in LE was increased on day 13 of P and decreased from day 10 to day 13 of C. This suggests a role of SOX17 in establishment of pregnancy but not directly in attachment of trophoblast cells to the LE.

The TF nuclear factor, interleukin 3 regulated (*NFIL3*) has been shown to be essential for the development of a subset of uterine natural killer (NK) cells secreting embryo growth-promoting factors in both humans and mice^61^. Furthermore, in rat granulosa cells, NFIL3 overexpression inhibited the induction of prostaglandin-endoperoxide synthase 2 (Ptgs2), progesterone receptor (Pgr), epiregulin (Ereg), and amphiregulin (Areg) and down-regulated levels of prostaglandin E2^62^. The upregulation of *NFIL3* in LE of P in both comparison 1 and 2 suggests a role in uterine NK cells and regulation of other key genes in equine endometrium.

A particularly pronounced transcript level upregulation in LE of P samples in both comparison 1 and 2 was obtained for the three nuclear receptor subfamily 4 group A members *NR4A1*, *NR4A2*, and *NR4A3*. Findings in human endometrium suggest a regulatory role of these TF also in the mare, leading to a “decidualization-like” differentiation state of the LE. Jiang et al.^63^ found *NR4A1* as essential for decidualization. NR4A receptors regulate forkhead box O1 (*FOXO1*) expression, which is mediating NR4A-induced decidualization^63^. Many decidualization-associated genes are NR4A-dependent via FOXO1, e.g., prolactin and *IGFBP1*. Upregulation of *FOXO1* (comparison 1) and *IGFBP1* (comparison 1 and 2) was found in LE of pregnant mares in the present study. Another role of NR4A1 has been described in PGR-driven endometrial vascular permeability where NR4A1 acts downstream of PGR in the regulation of the endothelial barrier function^64^. Several studies showed that members of the NR4A family are regulated by PGs. Prostaglandin F2 alpha and the PTGER2 agonist Butaprost have been shown to induce *NR4A1* expression in human embryonic kidney cells (HEK293)^65^. Prostaglandin E2 has been shown to upregulate *NR4A2* expression^66^. Furthermore, several studies showed that PGs are transactivating NR4A members by binding to these nuclear receptors, e.g., PGA2 binding to NR4A3^67^, and PGE1 and PGA1 binding to NR4A1^68^. The known roles of these TF in endometrium and the observed expression profiles in equine LE suggest a regulatory role in establishment of pregnancy controlled by conceptus PGs, which needs further mechanistic investigations.

In addition to the NR4A members, NR2F2 was found as upregulated in LE in comparison 1 and 2, adding it to the list of TF genes stimulated already by smaller conceptuses. The TF NR2F2 has been shown to mediate progesterone–Ihh signaling to regulate decidualization in mice^69^. In murine endometrium, stromal NR2F2 also promoted PGR expression to mediate P4-induced suppression of E2 activity in the epithelium^69^. It can be speculated that NR2F2 upregulation in equine LE is a species-specific mechanism to suppress E2 action and *ESR1* upregulation.

Although, the process of decidualization is restricted to animals with invasive implantation phenotypes^70^, a number of genes described as involved in decidualization in mice and human were found as DE in LE in the mare. In agreement with the role of stathmin-1 (*STMN1*) in the regulation of implantation and trophoblast invasion in the mouse and in humans^71,72^, the downregulation in equine LE of pregnant mares could be a way to prevent conceptus attachment and implantation. To facilitate an intact endometrial barrier and proper support of conceptus growth by endometrial secretions, regulation of cellular junctions is important. Adrenomedullin (*ADM*), which mRNA was increased in LE of pregnant vs. cyclic mares (significant in comparison 1 and tendency in comparison 2) has been shown to regulate water permeability and cell junctions in the epithelial and stromal compartments of the mouse uterus during peri-implantation time^73^. The DGE of members of the claudin family observed here could also be involved in this process, because claudins 3 and 10 showed different distribution patterns during decidualization and trophoblast invasion in mouse and human endometrium^74^, and CLDN4 has been suggested to be involved in regulation of water and ion transport through the LE^75^. Additional genes with known roles in decidualization and implantation were dual specificity phosphatase 1 (*DUSP1*), *EGR1*, integrin subunit beta 8 (*ITGB8*), and norrin cystine knot growth factor NDP (*NDP*)^76–79^. In agreement with the very late and non-invasive implantation in the mare, many genes known to have negative effects on decidualization and invasive implantation were found as upregulated in LE of pregnant compared to cyclic mares, e.g., parathyroid hormone-hike hormone (*PTHLH*)^80^, regulator of G protein signaling 2 (*RGS2*)^81^, and tissue inhibitor of metalloproteinases-3 (*TIMP3*)^82^.

In conclusion, the use of a spatiotemporal LCM-RNA-seq approach revealed the LE as the main player in the response to embryo signaling during the time of MRP. A very early response at the mRNA expression level in the LE was already found for a conceptus size of 3–4 mm in diameter. Overall, we identified a high number of DEGs and their potential upstream regulators in response to embryo signaling in LE, but also in GE and ST, pointing to key players in establishment of pregnancy. Specifically, data mining revealed regulation of PG transport and metabolism (synthesis and degradation, as well as several highly overrepresented TF families that could be involved in prevention of the release of luteolytic PGF2a and in establishing a receptive endometrium supporting conceptus growth and development. Finally, the results highlighted the uniqueness of establishment of pregnancy in equines at the gene expression level, pointing at key genes involved in epithelial barrier formation and prevention of conceptus attachment and implantation. Although our study is limited to the transcript level, since the small amount of material provided by the LCM makes it very difficult to perform proteomics analysis, it provides a broad basis for further functional studies. Ongoing experiments on the conceptus side (mRNA and proteins) and the uterine extracellular vesicles (and their molecular cargo) will add valuable information and help to unravel the molecules playing a key role in MRP in the mare.

## Methods

### Animal trial and sample collection

All methods were carried out in accordance with respective guidelines and regulations. The animal experiments were performed based on the permission of the Institutional Animal Care and Use Committee of the University of Illinois Urbana-Champaign (Protocol No.16129). The study was carried out in compliance with the ARRIVE guidelines (https://arriveguidelines.org/). Sample collection was performed with 14 light breed mares during 51 cycles of the 2018 breeding season of the northern hemisphere. The mares were housed on pasture at the Veterinary Medical Research Farm of the University of Illinois Urbana-Champaign (Illlinois, USA). The cycles (3–5 cycles) of each mare were randomly assigned to 6 groups: C days 10 and 13, and P days 10, 11, 12, 13 after ovulation. Follicular as well as CL development and ovulation were monitored (daily for the days before and after ovulation) with an Ibex® EVO® ultrasound device (E.I. Medical Imaging, Colorado, USA). When a follicle of at least 35 mm of diameter in combination with a uterine edema corresponding to estrus was detected, ovulation was induced (day – 2) with an intramuscular (i.m.) injection of 1.8 mg of deslorelin acetate (SucroMateTM Equine; Thorn BioScience L.L.C.; Kentucky, USA), a GnRH analogue. Mares assigned to a P group were inseminated 24 h later (day − 1) with 50 ml of fresh semen of the same fertile stallion extended with INRA96 (imv Technologies, France) (1:1). Mares assigned to a C group were inseminated with 50 ml of INRA96 24 h after ovulation induction. Uterine lavage was performed in all mares 6 h later with Lactated Ringer Solution (RLS) (Lactated Ringer’s Injection, USP; Hospira; Illinois, USA). Twenty International Units (IU) of oxytocin (VET ONE; Bimeda-MTC Animal Health Inc.; Cambridge, Canada) were administered i.m. immediately after uterine lavage as well as during the next 1–2 days (max. two times per day) to avoid intrauterine fluid accumulation. The day when ovulation was observed by transrectal ultrasonography (disappearance of the ovarian dominant follicle(s) and the identification of one (or two) CL) was designated as day 0. On sampling days (10, 11, 12, and 13), 100 ml of phosphate-buffered saline (PBS) (Corning cellgro; Virginia, USA) were transferred into the uterus using an equine uterine flushing catheter (8 mm inner and 10 mm outer diameter; JorgVet, Jorgensen Labs; Colorado, USA) in the C groups or an endotracheal tube for small ruminants (13.0 mm inner and 17.3 mm outer diameter; DEE Veterinary Products) for the P groups. The flush was then processed and stored accordingly for another study. If the conceptus was not already recovered in the small-volume uterine flush, a transcervical uterine flushing was performed with 1.5–2 l of RLS, repeating this procedure 1 to 3 times if necessary. The fluid was recovered into sterile glass bottles and passed through an embryo filter (Minitube, Germany). The recovered embryos were measured, photographed and immediately snap-frozen in liquid nitrogen. Endometrial samples were obtained by performing two trans-cervical uterine biopsies via an equine uterine biopsy forceps. The first biopsy was snap-frozen in liquid nitrogen and stored at − 80 °C until LCM and RNA-Seq. The second was fixated in 4% formalin and later embedded in paraffin for histological examination. For induction of luteolysis at the end of the experiment, 250 μg of cloprostenol, an analogue of PGF2a (Estrumate®; Merck Animal Health; Germany) was applied i.m. on the corresponding sampling day.

### Laser capture microdissection of target cells, RNA isolation and, RNA-Sequencing of endometrial biopsy samples

Frozen endometrial sample were embedded in optimal cutting temperature compound (OCT Compound; Biosystems, Switzerland) before performing LCM. Cryosections of 10 µm thickness were cut at -20 °C with a Leica CM1950 clinical Cryotome and mounted on 1.0 PEN NF Membrane Slides (Zeiss, Germany). Cresyl violet staining and LCM were performed as previously described^20,21^ to capture the target cells [luminal epithelium (LE), glandular epithelium (GE), and stroma including blood vessels (ST)] from the sections of each slide. In brief, a modified, rapid cresyl violet staining protocol was used to stain the sections and identify the different cell types of the endometrium. First, the slides were fixed for 2 min with 70% ethanol, and quickly washed in 50% ethanol. A 1% cresyl violet solution (Cresyl Violet acetate, C5042-10G; SIGMA; USA) was used to stain the tissue sections for 30 s, and then the slides were washed by dipping them 2–3 times in 50%, 70%, 100% ethanol, respectively. Finally, the slides were dried at room temperature. RNase-free water was used to prepare all solutions. To capture the target cells (LE, GE, and stroma) from the sections of each slide, a Zeiss 200 M inverse microscope Axiovert 40 (ZEISS PALM Microsystem, Zeiss, Germany) with a PALM MicroBeam, AxioCam Mrm camera, 355-nm pulsed UV laser, and PALM RoboSoftware v.4.6 (https://www.zeiss.com/microscopy/int/products/microscope-software/palm-robosoftware.html) was used. Once the target cells were cut satisfactory, the tissue pieces were lifted into adhesive caps (AdhesiveCap 200 clear, Zeiss). Each selected cell type was collected in a separate adhesive cap. Representative images of sections after dissecting LE and GE and collected samples visualized on a collection cap are shown in Fig. S2. Thereafter, the cells from the LCM samples were lysed by adding 50 µl extraction buffer (Arcturus™ PicoPure™ RNA Isolation Kit, Applied Biosystems, Vilnius, Lithuania) to the adhesive caps and incubation at 42 °C for 30 min. The lysates were then frozen on dry ice and stored at − 80 °C until RNA isolation. Total RNA was isolated from LE, GE and ST from each biopsy with the Arcturus™ PicoPure™ RNA Isolation Kit following the manufactures recommendations. Agilent RNA 6000 Pico assays (2100 Bioanalyzer, Agilent Technologies, Waldbronn, Germany) were used to assess quality and quantity of total RNA extracts from LE, GE, and ST samples. Samples showing a good quality (RIN > 6) were used for RNA-Seq library preparation starting from 2.5 ng total RNA and following the instructions of the Ovation® Solo Single Cell RNA Seq System kit (NuGen Technologies, San Carlos, USA). For the first amplification step, the number of PCR cycles was between 7 and 13. Each library was barcoded individually for multiplexing (5 different multiplex pools). Sequencing of the library pools was performed on in total 10 lanes of Illumina single-read flow cells on an Illumina HiSeq 2500 instrument (Functional Genomic Center Zurich).

### Data analysis

The resulting sequence reads (FastQ files) from the multiplex sequencing were analyzed with various specialized tools of a locally installed version of Galaxy^83^. Initially, the first 5 base pairs (bp) from the beginning of the reads (5′ end) were removed following the recommendations of the Ovation® Solo Single Cell RNA Seq System manual. The adapter sequence if present, as well as the low-quality ends (Quality Phred Score Cutoff = 28) from each read were trimmed. Before and after trimming, the quality of the Fastq files was checked with the FastQC-tool to confirm that the previous quality procedure was performed appropriately. The tool HISAT2 was used to perform the mapping of the sequences against the equine reference genome (EquCab3.0). Based on the 8 random bases (unique molecular identifier, UMI) contained in the barcode adapter of the libraries and the mapping coordinates in the BAM files, PCR duplicates were removed with the tool NUGEN NuDUP. A read count table was generated, and reads were filtered by a CPM cut-off before performing the statistical analysis using the Bioconductor package EdgeR (https://bioconductor.org/packages/edgeR/)^84^ for the identification of DEGs^84^. Genes with a false discovery rate (FDR) < 1% for LE (p-value < 0.0009), < 5% for GE (p-value < 0.0009), and < 13% for ST (p-value < 0.0009) were defined as DEGs. Known and putative human orthologous genes were assigned using an ortholog database^85^ in order to improve subsequent functional annotation analysis which was performed by the use of Metascape (www.metascape.org)^24^ and IPA software (https://digitalinsights.qiagen.com/products-overview/discovery-insights-portfolio/analysis-and-visualization/qiagen-ipa)^86^.

The RNA-seq data presented in this study are openly available at NCBI’s Sequence Read Archive (SRA) under the BioProject accession PRJNA748866.

## Supplementary Information

Supplementary Legends.

Supplementary Figures.

Supplementary Table S1.

Supplementary Table S2.

Supplementary Table S3.

Supplementary Table S4.

Supplementary Table S5.

Supplementary Table S6.

Supplementary Table S7.

Supplementary Table S8.

Supplementary Table S9.

Supplementary Table S10.
