# Supplementary material for: Spatiotemporal endometrial transcriptome analysis revealed the luminal epithelium as key player during initial maternal recognition of pregnancy in the mare
Source: Sci Rep. 2021 Nov 16;11:22293. doi: 10.1038/s41598-021-01785-3 (PMC8595723; doi:10.1038/s41598-021-01785-3)
Supplement: Supplementary file 1 — Supplementary Legends. [file 41598_2021_1785_MOESM1_ESM.docx]

## Supplementary information

***Supplemental Tables***

*Table S1.* Samples collected for pregnancy and control groups. Note that one mare (#20) is listed twice, since she was used twice (in two different cycles) for pregnancy day 12.

*Table S2:* Size and number of recovered conceptuses.

*Table S3:* Breed, age, and endometrial category in the scale of Kenney and Doig (1986) of each mare. Kenney and Doig categorized endometrial histological alterations into 4 different categories depending on the inflammatory, fibrotic, lymphatic, and atrophic changes found during histological evaluation. When more than one alteration is present, the category is lowered stepwise. Category I: degree of endometrial changes=absent, expected foaling rate=80-90%; Category IIA: degree of endometrial changes=mild, expected foaling rate=50-80%; Category IIB: degree of endometrial changes=moderate, expected foaling rate=10-50%; Category III: degree of endometrial changes=severe, expected foaling rate=10%).

*Table S4:* Results of RNA-sequencing for all detectable genes in luminal epithelium in comparison of samples derived from pregnant and cyclic control mares. LE comparison 1: EdgeR results for larger conceptuses vs. cyclic controls; LE comparison 2: EdgeR results for smaller conceptuses vs. cyclic controls; LE C13 vs. C10: differentially expressed genes between C13 and C10 (FDR 5%).

*Table S5:* Results of RNA-sequencing for all detectable genes in glandular epithelium in comparison of samples derived from pregnant and cyclic control mares. GE comparison 1: EdgeR results for larger conceptuses vs. cyclic controls; GE comparison 2: EdgeR results for smaller conceptuses vs. cyclic controls.

*Table S6:* Results of RNA-sequencing for all detectable genes in stroma in comparison of samples derived from pregnant and cyclic control mares. ST comparison 1: EdgeR results for larger conceptuses vs. cyclic controls; ST comparison 2: EdgeR results for smaller conceptuses vs. cyclic controls.

*Table S7:* Results of Metascape functional term overrepresentation analysis for genes up- or downregulated in luminal epithelium, glandular epithelium, and stroma.

*Table S8:* Overlap of DEGs in LE of comparison 1 (larger conceptuses vs. cyclic controls), comparison 2 (smaller conceptuses vs. cyclic controls), and comparison of C13 vs. C10.

*Table S9:* Results of Metascape membership analysis for genes up- or downregulated in luminal epithelium.

*Table S10:* Genes identified by self-organizing tree algorithm (SOTA clustering) with expression in LE either decreasing from day 10 to day 13 of the estrous cycle in LE but unchanged or upregulated in pregnancy samples (“P13 up”) or increasing from day 10 to day 13 of the estrous cycle but unchanged or downregulated in pregnancy samples (“P13 down”).

***Supplemental Figures***

*Figure S1:* Experimental design and workflow of the study. Image created with PALM RoboSoftware v.4.6 (https://www.zeiss.com/microscopy/int/products/microscope-software/palm-robosoftware.html) and Microsoft® Microsoft 365 PowerPoint (https://www.microsoft.com/en-us/microsoft-365/powerpoint).

*Figure S2:* Laser capture microdissection (LCM) using a Zeiss PALM MicroBeam instrument of frozen cresyl-violet-stained endometrial biopsies. **A** Endometrial section before LCM. **B** Endometrial section after dissecting luminal epithelium (LE) and glandular epithelium (GE). **C** Endometrial section after dissecting LE, GE and ST. **D-F** The different LCM sample types visualized on a collection cap after the isolation of LE, GE, and ST samples (from D to F). Image created with PALM RoboSoftware v.4.6 (https://www.zeiss.com/microscopy/int/products/microscope-software/palm-robosoftware.html) and modified with Adobe Photoshop v.22.4.3.

*Figure S3:* Hierarchical cluster analysis of differentially expressed genes (FDR <1%) obtained for luminal epithelium (LE) when comparing all LE samples from pregnant and cyclic mares. The color scale illustrates log2 mean-centered expression values with higher expression than the mean of all samples shown in red and lower expression than the mean of all samples shown in blue. Image created with the R package gplots v.3.1.1 (https://CRAN.R-project.org/package=gplots) and modified with Adobe Photoshop v.22.4.3.

*Figure S4:* Hierarchical cluster analysis of the differentially expressed genes (DEGs) obtained for each cell type when comparing samples from pregnant and cyclic mares. The color scale illustrates log2 mean-centered expression values with higher expression than the mean of all samples shown in red and lower expression than the mean of all samples shown in blue. **A** Luminal epithelium (LE) with 1253 DEGs (FDR <1%, p-value <0.0009). **B** Glandular epithelium (GE) with 248 DEGs (FDR<5%, p-value <0.0009). **C** Stroma (ST) with 103 DEGs (FDR<13%, p-value <0.0009). Sample names: day of cycle (C) or pregnancy (P) followed by sample number and diameter of recovered conceptuses in mm (for P samples). Image created with Multiple Experiment Viewer (MeV v.4.8.1, https://sourceforge.net/projects/mev-tm4/)^1^ and modified with Adobe Photoshop v.22.4.3.

*Figure S5:* Clustering of luminal epithelium (LE) samples derived from pregnant and cyclic mares. Self-organizing Map (SOM) analysis was performed for the differentially expressed genes (FDR <1%) identified in LE resulting in 5 clusters of samples. Expression graphs of the clusters are shown in the upper part. The lower part shows the expression images. Day of cycle/pregnancy and size of conceptus(es) are shown. The color scale illustrates the log2-mean centered expression values (log2 counts per million of the sample minus mean of all samples) from blue (-4) over white (0) to red (+4). Image created with Multiple Experiment Viewer (MeV v.4.8.1, https://sourceforge.net/projects/mev-tm4/)^1^ and modified with Adobe Photoshop v.22.4.3.

*Figure S6:* Comparative functional enrichment analysis of the differentially expressed genes (comparison 1) in luminal epithelium (LE), glandular epithelium (GE), and stroma (ST). Metascape tool and IPA software were used to identify enriched functional terms, biological processes, and canonical pathways. **A** Heatmap of the top 100 functional terms obtained by Metascape. Terms are colored by statistical significance (-log10 of P-value) from gray (not significant) to brown (highly significant). **B** Overrepresented disease and biological function categories revealed by IPA software (Z-score ≥2 and log10 (P-value) ≥2). Dots in the heatmap indicate log10 (P-value) <2. **C** Overrepresented canonical pathways revealed by IPA software (Z-score ≥1 and log10 (P-value) ≥2). Dots in the heatmap indicate log10 (P-value) <2. Image created with Metascape webtool (https://metascape.org)^2^ and Ingenuity Pathway Analysis software v.68752261 (https://digitalinsights.qiagen.com/products-overview/discovery-insights-portfolio/analysis-and-visualization/qiagen-ipa)^3^, and modified with Adobe Photoshop v.22.4.3.

*Figure S7:* Upstream regulator analysis of the differentially expressed genes (comparison 1) in luminal epithelium (LE), glandular epithelium (GE), and stroma (ST). **A** Results obtained with Ingenuity Pathway Analysis (IPA) software (Z-score ≥2 and log10 (P-value) ≥2). **B** Results obtained with Metascape tool (color scale represents statistical significance, –log10(P): –log10 of P-value). Image created with Metascape webtool (https://metascape.org)^2^ and Ingenuity Pathway Analysis software v.68752261 (https://digitalinsights.qiagen.com/products-overview/discovery-insights-portfolio/analysis-and-visualization/qiagen-ipa)^3^, and modified with Adobe Photoshop v.22.4.3.

*Figure S8:* Comparison of target genes between different potential upstream regulators obtained for the differentially expressed genes in luminal epithelium (comparison 1) by Ingenuity Pathway Analysis (IPA) software. Multiple Venn diagrams were performed for the target genes of beta-estradiol (E2), epidermal growth factor (EGF), transforming growth factor beta (TGFB), tumor necrosis factor (TNF), interferon gamma (IFNG), interleukin 1 beta (IL1B), prostaglandin F 2 alpha (PGF2a), leukotriene D4 (LTD4), choriogonadotropin (CG) complex, insulin-like growth factor 1 (IGF1), oleic acid, sphingosine, and aldosterone. Image created with jvenn webtool (http://jvenn.toulouse.inra.fr/app/example.html)^4^ and modified with Adobe Photoshop v.22.4.3.

*Figure S9:* Selected upstream regulator networks obtained for the differentially expressed genes in luminal epithelium (comparison 1) by IPA software. Master regulators: beta-estradiol, epidermal growth factor (EGF), transforming growth factor beta 1 (TGFB1), cAMP responsive element binding protein 1 (CREB1), prostaglandin F 2 alpha (PGF2a), leukotriene D4, interferon gamma (IFNG), tumor necrosis factor (TNF), interleukin 1 beta (IL1B), aldosterone, sphingosine-1-phosphate, and oleic acid. Image created with Ingenuity Pathway Analysis software v.68752261 (https://digitalinsights.qiagen.com/products-overview/discovery-insights-portfolio/analysis-and-visualization/qiagen-ipa)^3^ and modified with Adobe Photoshop v.22.4.3.

*Figure S10:* Comparative functional enrichment and upstream regulator analysis of the differentially expressed genes (DEGs) in luminal epithelium (LE) between comparisons 1 and 2. **A** Heatmap of overrepresented functional terms and pathways obtained by Metascape tool. **B** Overrepresented disease and biological function categories revealed by Ingenuity Pathway Analysis (IPA) software (Z-score ≥2 and log10 (P-value) ≥2). Dots in the heatmap indicate Z-score <2. **C** Overrepresented canonical pathways revealed by IPA software (Z-score ≥1.5 and log10 (P-value) ≥2). Dots in the heatmap indicate log10 (P-value) <1.5. **D** Upstream regulator analysis results obtained with Metascape tool (color scale represents statistical significance, –log10(P): –log10 of P-value). **E** Upstream regulator analysis results obtained with IPA (Z-score ≥2 and log10 (P-value) ≥2). C1: comparison 1; C2: comparison 2; HP: higher expression in pregnancy samples, LP: lower expression in pregnancy samples. Image created with Metascape webtool (https://metascape.org)^2^ and Ingenuity Pathway Analysis software v.68752261 (https://digitalinsights.qiagen.com/products-overview/discovery-insights-portfolio/analysis-and-visualization/qiagen-ipa)^3^, and modified with Adobe Photoshop v.22.4.3.

*Figure S11:* Clustering of genes with similar expression profiles across cycle and pregnancy days. Mean log2 counts per million (cpm) values were calculated for each day of the estrous cycle and pregnancy for genes differentially expressed in LE between pregnant and cyclic samples (comparison 1, FDR 1%) and between day 13 and day 10 of the estrous cycle (FDR 5%). The log2 cpm values per day were used to calculate mean-centered expression values (log2 cpm of the sample minus mean of all samples). Self-organizing tree algorithm analysis (Multiple Experiment Viewer, MeV)^1^ was performed to identify clusters of genes with similar expression profiles. The number in the top left corner of each graph represents the number of genes in the cluster. C10/C13: days 10/13 of cycle; P10-P13: days 10 to 13 of pregnancy. Vertical axis: mean-centered expression values in log2 scale. Image created with Multiple Experiment Viewer (MeV v.4.8.1, https://sourceforge.net/projects/mev-tm4/)^1^ and modified with Adobe Photoshop v.22.4.3.

*Figure S12:* Overrepresented functional categories for differentially expressed genes (DEGs) in luminal epithelium (LE) between cycle day 13 and 10. Genes of the clusters shown in Figure 4 were selected based on the expression differences between days 13 of P and day 13 of C (log2 FC≥1) to obtain genes which expression was either decreasing from day 10 to day 13 of the cycle but unchanged in pregnancy samples (clusters 3 and 4, “P13 up”) or increasing from day 10 to day 13 of the cycle but unchanged in pregnancy samples (clusters 5 and 6, “P13 down”). Metascape analysis^2^ was performed for the corresponding gene lists. The top 100 overrepresented functional terms, biological processes, and canonical pathways are shown. The heatmaps are colored by statistical significance (–log10 of P-value) from gray (not significant) to brown (highly significant). Image created with Metascape webtool (https://metascape.org)^2^ and modified with Adobe Photoshop v.22.4.3.

*Figure S13:* Regulatory interaction network of nuclear receptor subfamily 2 group F member 2 (NR2F2). Ingenuity Pathway Analysis (IPA) software was used to generate a regulatory interaction network for NR2F2. For genes detected as expressed in luminal epithelium, log2 fold change is shown in the bar plot next to the gene symbol for pregnancy vs. cyclic control and pregnancy day 13 vs. cycle day 13. Image created with Ingenuity Pathway Analysis software v.68752261 (https://digitalinsights.qiagen.com/products-overview/discovery-insights-portfolio/analysis-and-visualization/qiagen-ipa)^3^ and modified with Adobe Photoshop v.22.4.3.

**References**

1 Howe, E. A., Sinha, R., Schlauch, D. & Quackenbush, J. RNA-Seq analysis in MeV. *Bioinformatics* **27**, 3209-3210, doi:10.1093/bioinformatics/btr490 (2011).

2 Zhou, Y. *et al.* Metascape provides a biologist-oriented resource for the analysis of systems-level datasets. *Nat Commun* **10**, 1523, doi:10.1038/s41467-019-09234-6 (2019).

3 Kramer, A., Green, J., Pollard, J., Jr. & Tugendreich, S. Causal analysis approaches in Ingenuity Pathway Analysis. *Bioinformatics* **30**, 523-530, doi:10.1093/bioinformatics/btt703 (2014).

4 Bardou, P., Mariette, J., Escudie, F., Djemiel, C. & Klopp, C. jvenn: an interactive Venn diagram viewer. *BMC Bioinformatics* **15**, 293, doi:10.1186/1471-2105-15-293 (2014).
