# Supplementary material for: Spatiotemporal endometrial transcriptome analysis revealed the luminal epithelium as key player during initial maternal recognition of pregnancy in the mare
Source: Sci Rep. 2021 Nov 16;11:22293. doi: 10.1038/s41598-021-01785-3 (PMC8595723; doi:10.1038/s41598-021-01785-3)
Supplement: Supplementary file 2 — Supplementary Figures. [file 41598_2021_1785_MOESM2_ESM.pdf]

Figure S1

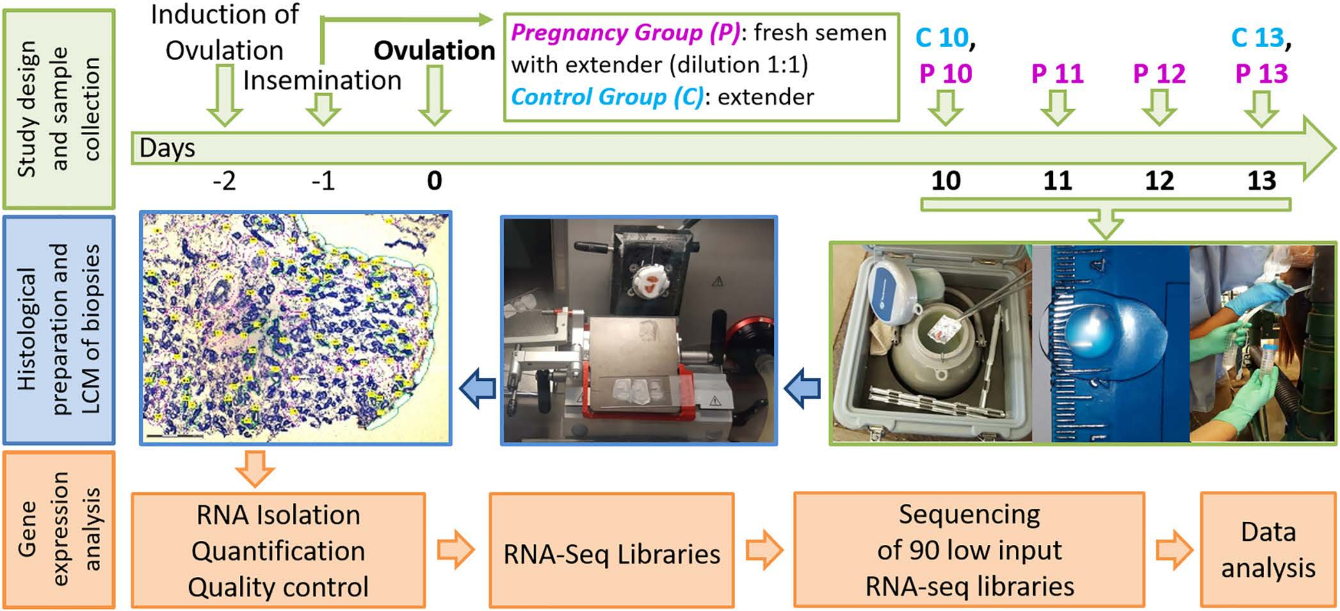
Study design and sample collection
Induction of Ovulation
Insemination
Ovulation
Pregnancy Group (P): fresh semen with extender (dilution 1:1)
Control Group (C): extender
C 10, P 10
P 11
P 12
C 13, P 13
Days
-2
-1
0
10
11
12
13
Histological preparation and LCM of biopsies
Gene expression analysis
RNA Isolation
Quantification
Quality control
RNA-Seq Libraries
Sequencing of 90 low input RNA-seq libraries
Data analysis

Figure S2

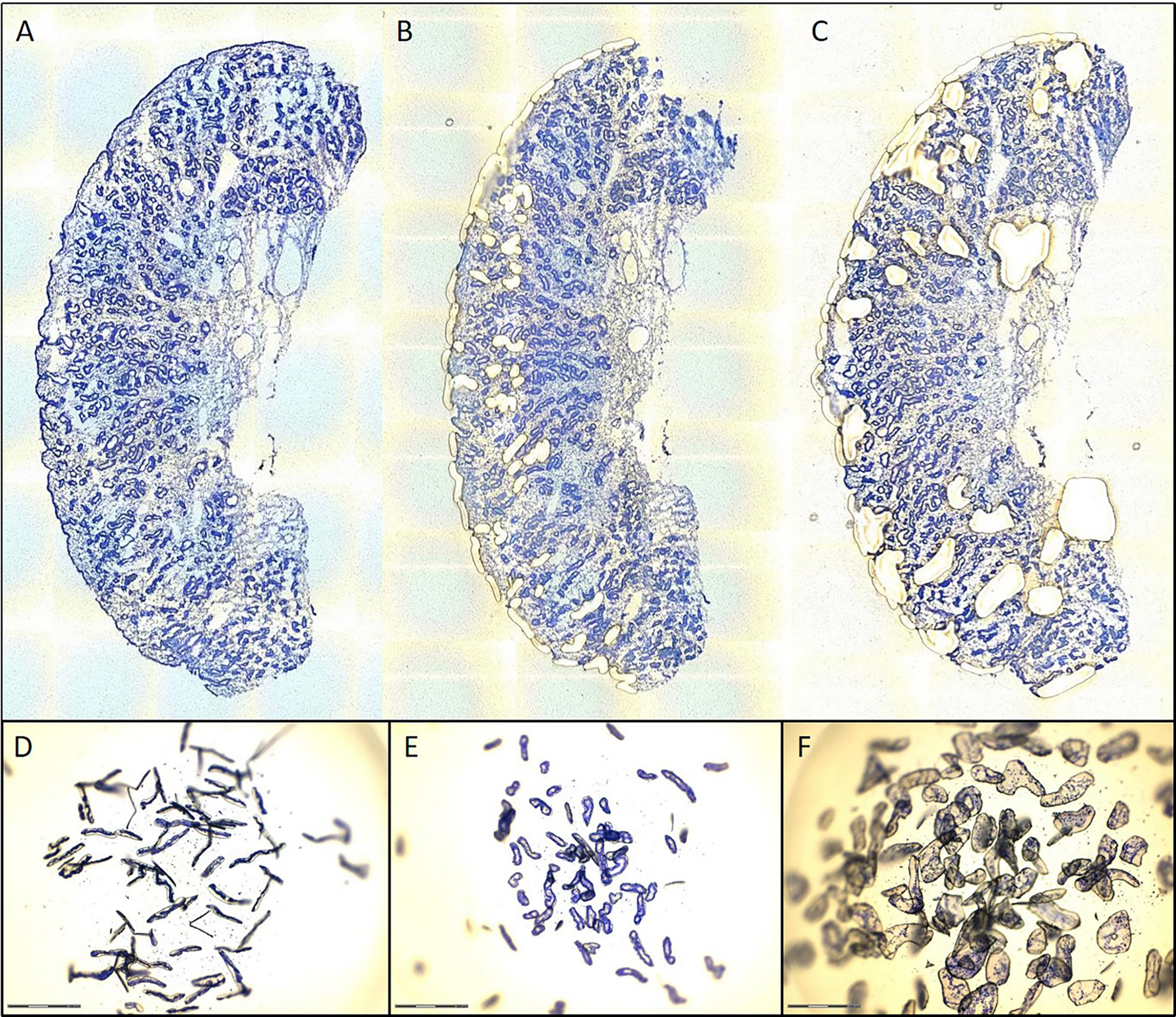

Figure S3

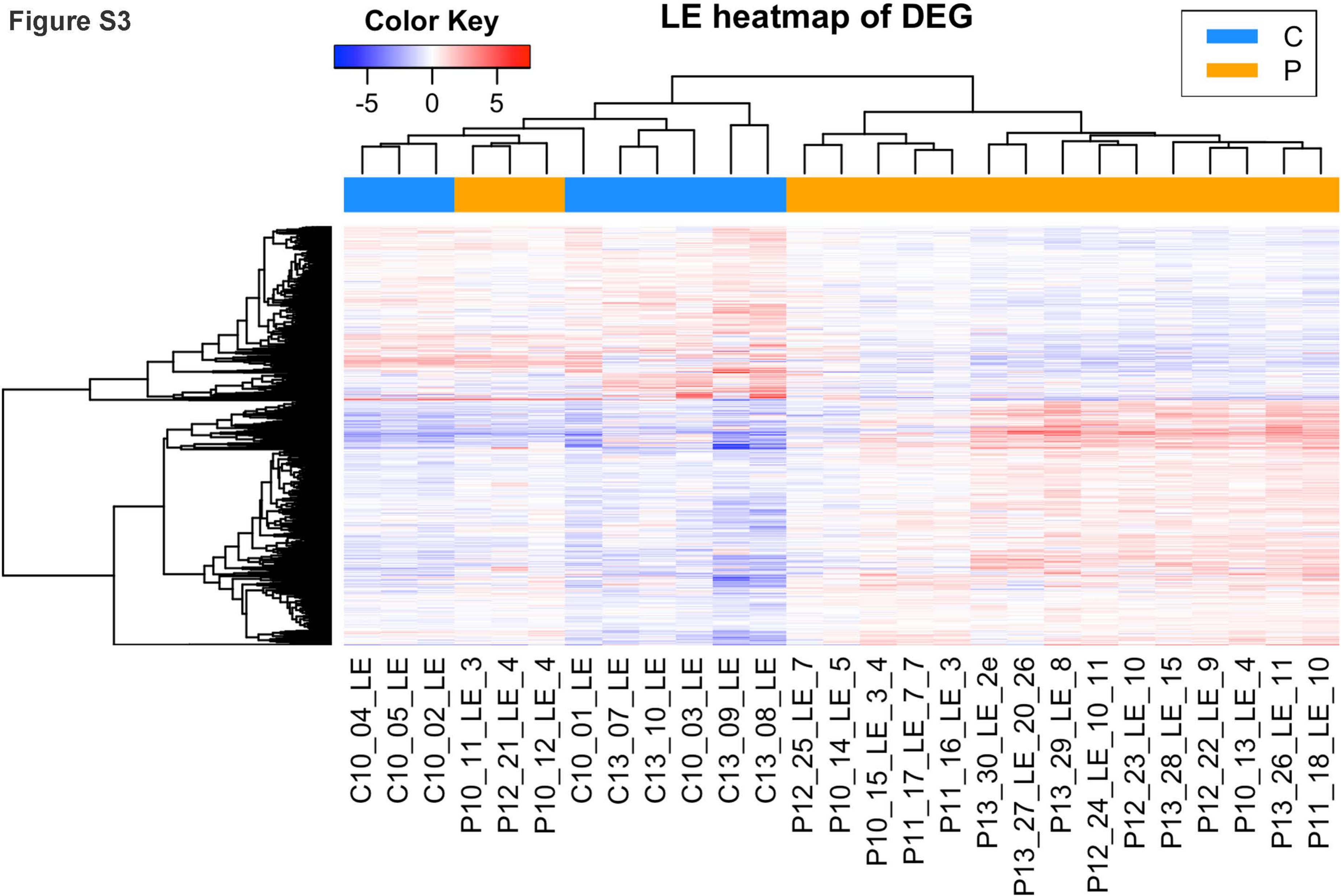

Color Key
-5
0
5
LE heatmap of DEG
C
P
C10_04_LE
C10_05_LE
C10_02_LE
P10_11_LE_3
P12_21_LE_4
P10_12_LE_4
C10_01_LE
C13_07_LE
C13_10_LE
C10_03_LE
C13_09_LE
C13_08_LE
P12_25_LE_7
P10_14_LE_5
P10_15_LE_3_4
P11_17_LE_7_7
P11_16_LE_3
P13_30_LE_2e
P13_27_LE_20_26
P13_29_LE_8
P12_24_LE_10_11
P12_23_LE_10
P13_28_LE_15
P12_22_LE_9
P10_13_LE_4
P13_26_LE_11
P11_18_LE_10

Figure S4

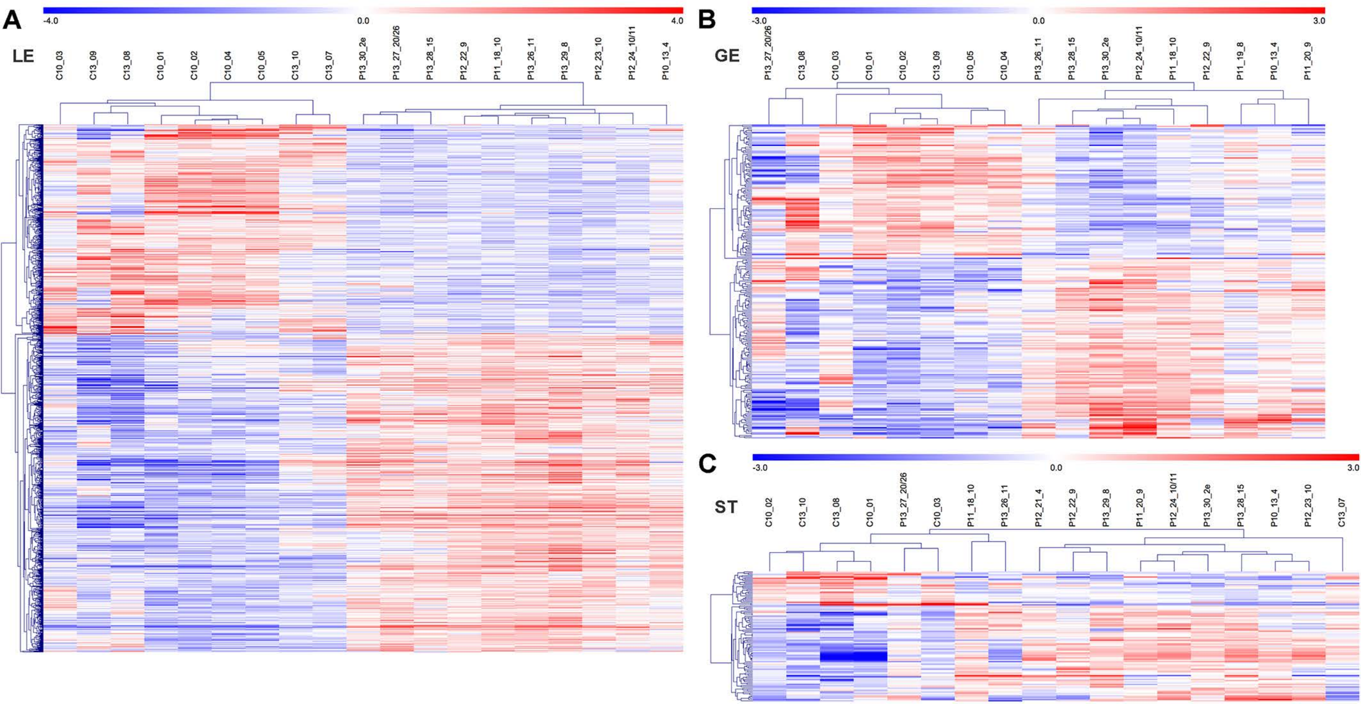
A
LE
-4.0
0.0
4.0
C10_03
C13_09
C13_08
C10_01
C10_02
C10_04
C10_05
C13_10
C13_07
P13_30_2e
P13_27_20/26
P13_28_15
P12_22_9
P11_18_10
P13_26_11
P13_29_8
P12_23_10
P12_24_10/11
P10_13_4
B
GE
-3.0
0.0
3.0
P13_27_20/26
C13_08
C10_03
C10_01
C10_02
C13_09
C10_05
C10_04
P13_26_11
P13_28_15
P13_30_2e
P12_24_10/11
P11_18_10
P12_22_9
P11_19_8
P10_13_4
P11_20_9
C
ST
-3.0
0.0
3.0
C10_02
C13_10
C13_08
C10_01
P13_27_20/26
C10_03
P11_18_10
P13_26_11
P12_21_4
P12_22_9
P13_29_8
P11_20_9
P12_24_10/11
P13_30_2e
P13_28_15
P10_13_4
P12_23_10
C13_07

Figure S5

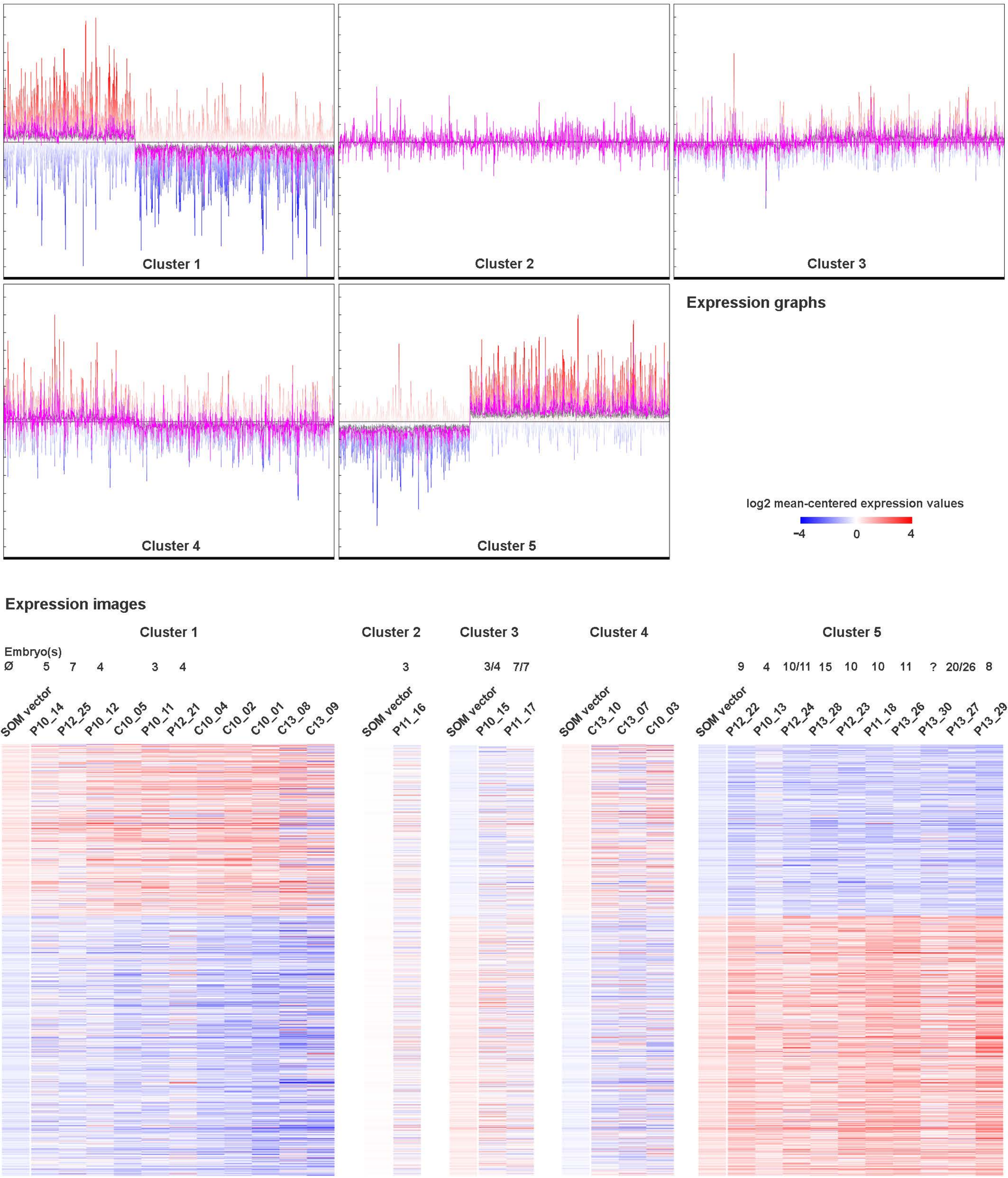
Cluster 1
Cluster 2
Cluster 3
Cluster 4
Cluster 5
Expression graphs
log2 mean-centered expression values
−4
0
4
Expression images
Cluster 1
Cluster 2
Cluster 3
Cluster 4
Cluster 5
Embryo(s)
Ø 5 7 4 3 4
3
3/4 7/7
9 4 10/11 15 10 10 11 ? 20/26 8
SOM vector P10_14 P12_25 P10_12 C10_05 P10_11 P12_21 C10_04 C10_02 C10_01 C13_08 C13_09
SOM vector P11_16
SOM vector P10_15 P11_17
SOM vector C13_10 C13_07 C10_03
SOM vector P12_22 P10_13 P12_24 P13_28 P12_23 P11_18 P13_26 P13_30 P13_27 P13_29

Figure S6

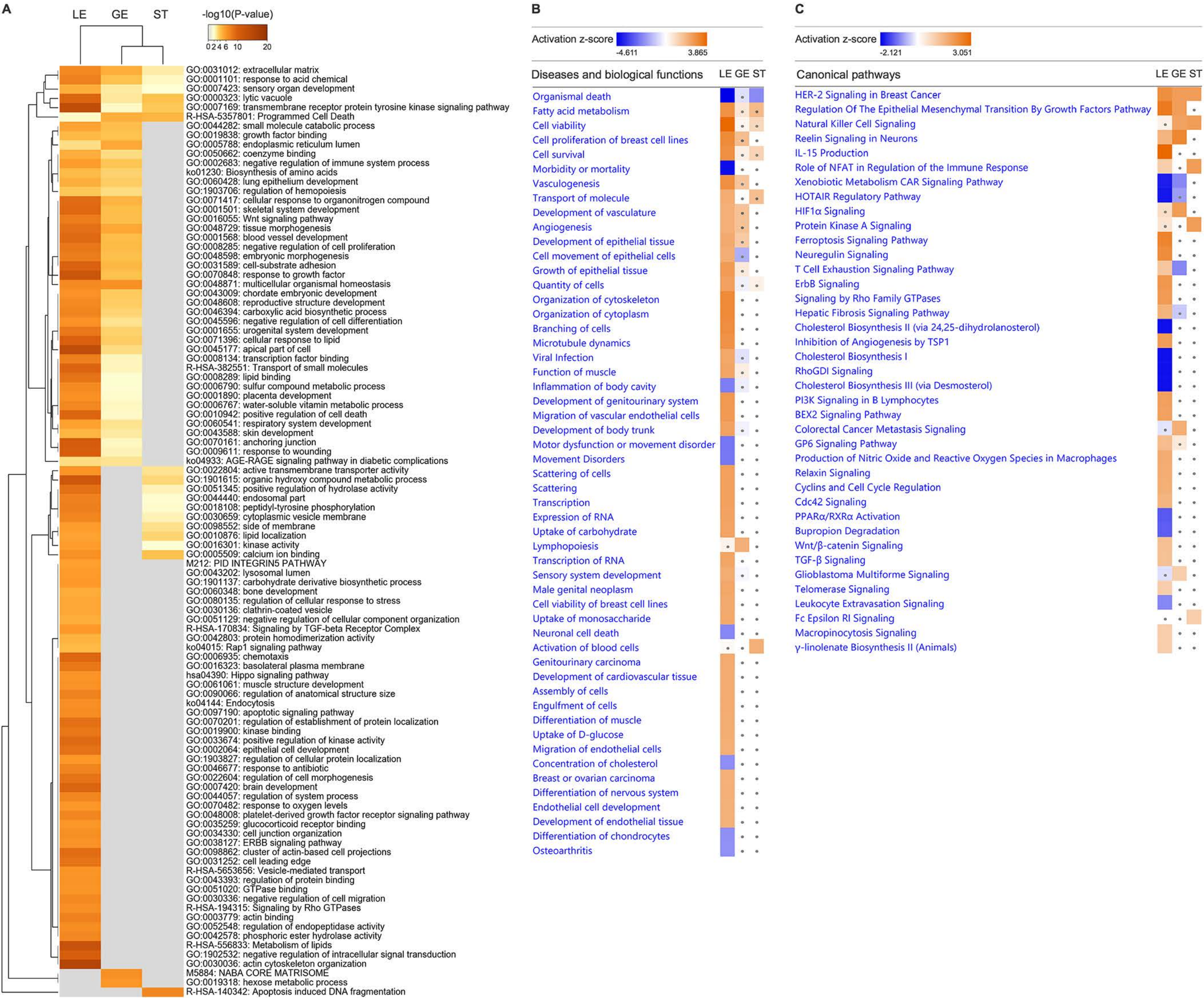
A
LE
GE
ST
-log10(P-value)
0 2 4 6 10 20
GO:0031012: extracellular matrix
GO:0001101: response to acid chemical
GO:0007423: sensory organ development
GO:0000323: lytic vacuole
GO:0007169: transmembrane receptor protein tyrosine kinase signaling pathway
R-HSA-5357801: Programmed Cell Death
GO:0044282: small molecule catabolic process
GO:0019838: growth factor binding
GO:0005788: endoplasmic reticulum lumen
GO:0050662: coenzyme binding
GO:0002683: negative regulation of immune system process
ko01230: Biosynthesis of amino acids
GO:0060428: lung epithelium development
GO:1903706: regulation of hemopoiesis
GO:0071417: cellular response to organonitrogen compound
GO:0001501: skeletal system development
GO:0016055: Wnt signaling pathway
GO:0048729: tissue morphogenesis
GO:0001568: blood vessel development
GO:0008285: negative regulation of cell proliferation
GO:0048598: embryonic morphogenesis
GO:0031589: cell-substrate adhesion
GO:0070848: response to growth factor
GO:0048871: multicellular organismal homeostasis
GO:0043009: chordate embryonic development
GO:0048608: reproductive structure development
GO:0046394: carboxylic acid biosynthetic process
GO:0045596: negative regulation of cell differentiation
GO:0001655: urogenital system development
GO:0071396: cellular response to lipid
GO:0045177: apical part of cell
GO:0008134: transcription factor binding
R-HSA-382551: Transport of small molecules
GO:0008289: lipid binding
GO:0006790: sulfur compound metabolic process
GO:0001890: placenta development
GO:0006767: water-soluble vitamin metabolic process
GO:0010942: positive regulation of cell death
GO:0060541: respiratory system development
GO:0043588: skin development
GO:0070161: anchoring junction
GO:0009611: response to wounding
ko04933: AGE-RAGE signaling pathway in diabetic complications
GO:0022804: active transmembrane transporter activity
GO:1901615: organic hydroxy compound metabolic process
GO:0051345: positive regulation of hydrolase activity
GO:0044440: endosomal part
GO:0018108: peptidyl-tyrosine phosphorylation
GO:0030659: cytoplasmic vesicle membrane
GO:0098552: side of membrane
GO:0010876: lipid localization
GO:0016301: kinase activity
GO:0005509: calcium ion binding
M212: PID INTEGRIN5 PATHWAY
GO:0043202: lysosomal lumen
GO:1901137: carbohydrate derivative biosynthetic process
GO:0060348: bone development
GO:0080135: regulation of cellular response to stress
GO:0030136: clathrin-coated vesicle
GO:0051129: negative regulation of cellular component organization
R-HSA-170834: Signaling by TGF-beta Receptor Complex
GO:0042803: protein homodimerization activity
ko04015: Rap1 signaling pathway
GO:0006935: chemotaxis
GO:0016323: basolateral plasma membrane
hsa04390: Hippo signaling pathway
GO:0061061: muscle structure development
GO:0090066: regulation of anatomical structure size
ko04144: Endocytosis
GO:0097190: apoptotic signaling pathway
GO:0070201: regulation of establishment of protein localization
GO:0019900: kinase binding
GO:0033674: positive regulation of kinase activity
GO:0002064: epithelial cell development
GO:1903827: regulation of cellular protein localization
GO:0046677: response to antibiotic
GO:0022604: regulation of cell morphogenesis
GO:0007420: brain development
GO:0044057: regulation of system process
GO:0070482: response to oxygen levels
GO:0048008: platelet-derived growth factor receptor signaling pathway
GO:0035259: glucocorticoid receptor binding
GO:0034330: cell junction organization
GO:0038127: ERBB signaling pathway
GO:0098862: cluster of actin-based cell projections
GO:0031252: cell leading edge
R-HSA-5653656: Vesicle-mediated transport
GO:0043393: regulation of protein binding
GO:0051020: GTPase binding
GO:0030336: negative regulation of cell migration
R-HSA-194315: Signaling by Rho GTPases
GO:0003779: actin binding
GO:0052548: regulation of endopeptidase activity
GO:0042578: phosphoric ester hydrolase activity
R-HSA-556833: Metabolism of lipids
GO:1902532: negative regulation of intracellular signal transduction
GO:0030036: actin cytoskeleton organization
M5884: NABA CORE MATRISOME
GO:0019318: hexose metabolic process
R-HSA-140342: Apoptosis induced DNA fragmentation
B
Activation z-score
-4.611
3.865
Diseases and biological functions
LE GE ST
Organismal death
Fatty acid metabolism
Cell viability
Cell proliferation of breast cell lines
Cell survival
Morbidity or mortality
Vasculogenesis
Transport of molecule
Development of vasculature
Angiogenesis
Development of epithelial tissue
Cell movement of epithelial cells
Growth of epithelial tissue
Quantity of cells
Organization of cytoskeleton
Organization of cytoplasm
Branching of cells
Microtubule dynamics
Viral Infection
Function of muscle
Inflammation of body cavity
Development of genitourinary system
Migration of vascular endothelial cells
Development of body trunk
Motor dysfunction or movement disorder
Movement Disorders
Scattering of cells
Scattering
Transcription
Expression of RNA
Uptake of carbohydrate
Lymphopoiesis
Transcription of RNA
Sensory system development
Male genital neoplasm
Cell viability of breast cell lines
Uptake of monosaccharide
Neuronal cell death
Activation of blood cells
Genitourinary carcinoma
Development of cardiovascular tissue
Assembly of cells
Engulfment of cells
Differentiation of muscle
Uptake of D-glucose
Migration of endothelial cells
Concentration of cholesterol
Breast or ovarian carcinoma
Differentiation of nervous system
Endothelial cell development
Development of endothelial tissue
Differentiation of chondrocytes
Osteoarthritis
C
Activation z-score
-2.121
3.051
Canonical pathways
LE GE ST
HER-2 Signaling in Breast Cancer
Regulation Of The Epithelial Mesenchymal Transition By Growth Factors Pathway
Natural Killer Cell Signaling
Reelin Signaling in Neurons
IL-15 Production
Role of NFAT in Regulation of the Immune Response
Xenobiotic Metabolism CAR Signaling Pathway
HOTAIR Regulatory Pathway
HIF1α Signaling
Protein Kinase A Signaling
Ferroptosis Signaling Pathway
Neuregulin Signaling
T Cell Exhaustion Signaling Pathway
ErbB Signaling
Signaling by Rho Family GTPases
Hepatic Fibrosis Signaling Pathway
Cholesterol Biosynthesis II (via 24,25-dihydrolanosterol)
Inhibition of Angiogenesis by TSP1
Cholesterol Biosynthesis I
RhoGDI Signaling
Cholesterol Biosynthesis III (via Desmosterol)
PI3K Signaling in B Lymphocytes
BEX2 Signaling Pathway
Colorectal Cancer Metastasis Signaling
GP6 Signaling Pathway
Production of Nitric Oxide and Reactive Oxygen Species in Macrophages
Relaxin Signaling
Cyclins and Cell Cycle Regulation
Cdc42 Signaling
PPARα/RXRα Activation
Bupropion Degradation
Wnt/β-catenin Signaling
TGF-β Signaling
Glioblastoma Multiforme Signaling
Telomerase Signaling
Leukocyte Extravasation Signaling
Fc Epsilon RI Signaling
Macropinocytosis Signaling
γ-linolenate Biosynthesis II (Animals)

Figure S7

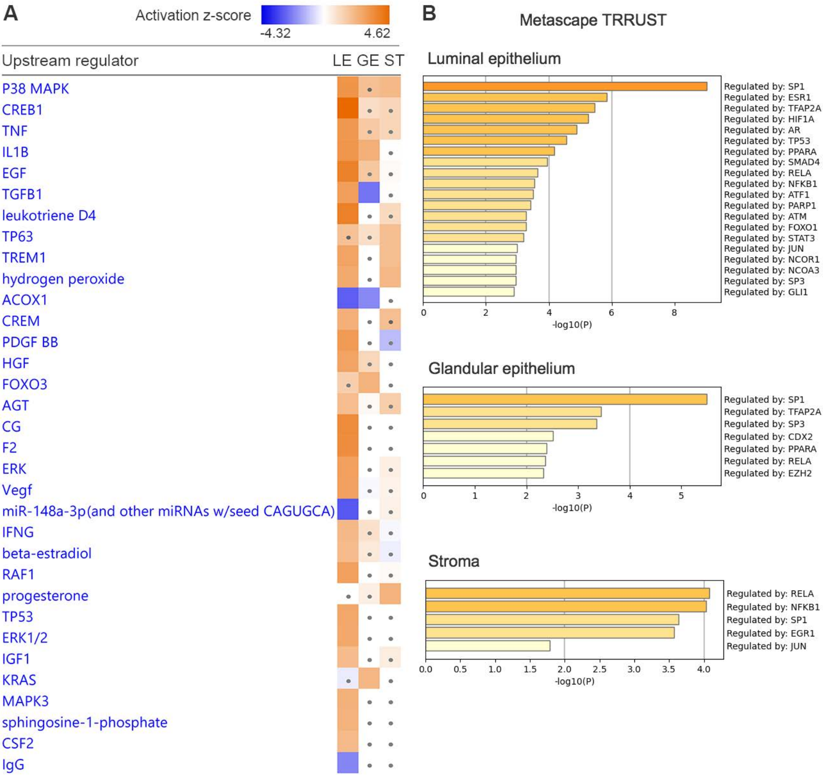
A
Activation z-score
-4.32
4.62
Upstream regulator
LE GE ST
P38 MAPK
CREB1
TNF
IL1B
EGF
TGFB1
leukotriene D4
TP63
TREM1
hydrogen peroxide
ACOX1
CREM
PDGF BB
HGF
FOXO3
AGT
CG
F2
ERK
Vegf
miR-148a-3p(and other miRNAs w/seed CAGUGCA)
IFNG
beta-estradiol
RAF1
progesterone
TP53
ERK1/2
IGF1
KRAS
MAPK3
sphingosine-1-phosphate
CSF2
IgG
B
Metascape TRRUST
Luminal epithelium
Regulated by: SP1
Regulated by: ESR1
Regulated by: TFAP2A
Regulated by: HIF1A
Regulated by: AR
Regulated by: TP53
Regulated by: PPARA
Regulated by: SMAD4
Regulated by: RELA
Regulated by: NFKB1
Regulated by: ATF1
Regulated by: PARP1
Regulated by: ATM
Regulated by: FOXO1
Regulated by: STAT3
Regulated by: JUN
Regulated by: NCOR1
Regulated by: NCOA3
Regulated by: SP3
Regulated by: GLI1
0 2 4 6 8
-log10(P)
Glandular epithelium
Regulated by: SP1
Regulated by: TFAP2A
Regulated by: SP3
Regulated by: CDX2
Regulated by: PPARA
Regulated by: RELA
Regulated by: EZH2
0 1 2 3 4 5
-log10(P)
Stroma
Regulated by: RELA
Regulated by: NFKB1
Regulated by: SP1
Regulated by: EGR1
Regulated by: JUN
0.0 0.5 1.0 1.5 2.0 2.5 3.0 3.5 4.0
-log10(P)

Figure S8

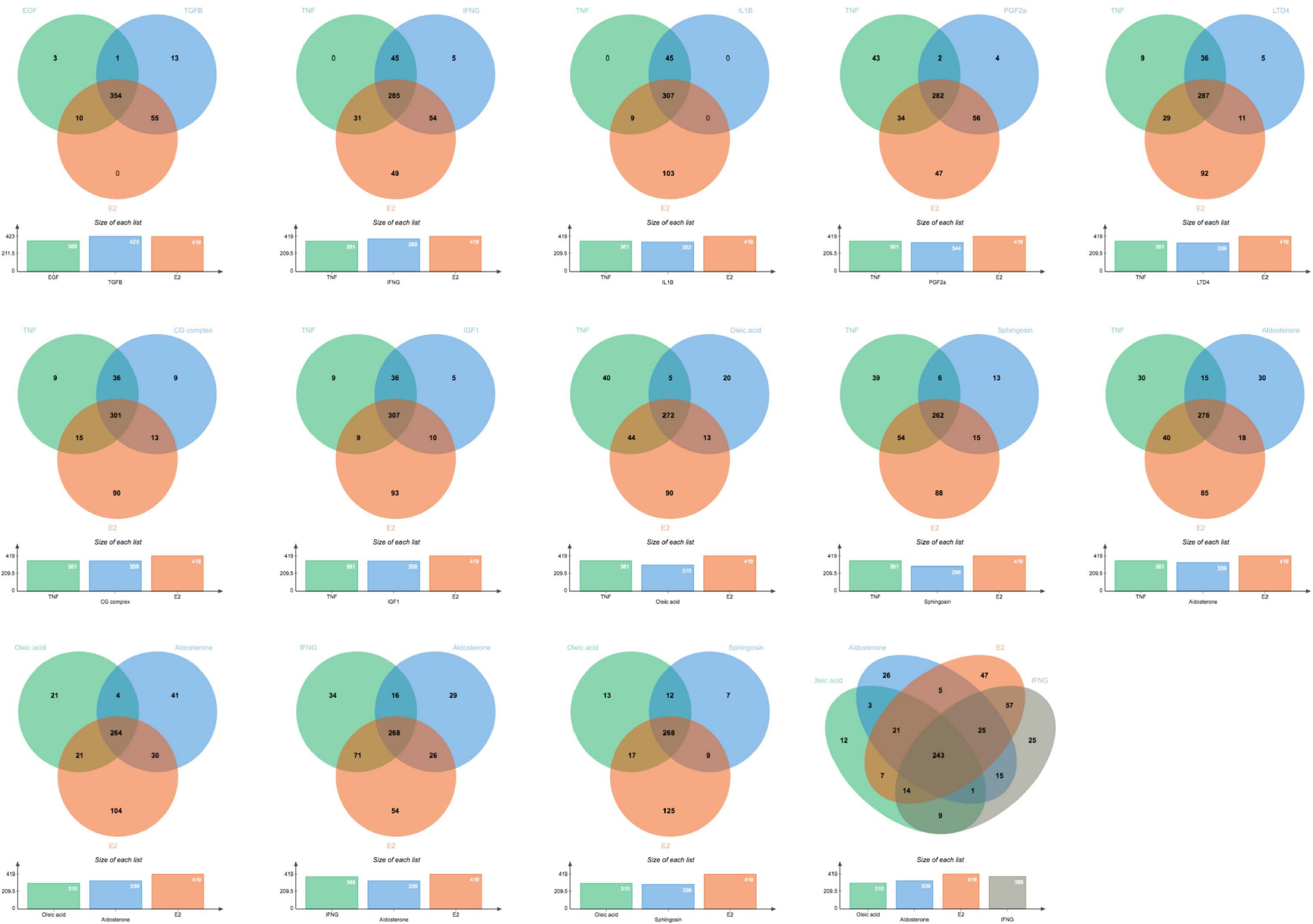
EGF
TGFB
3
1
13
354
10
55
0
E2
Size of each list
423
211.5
0
368
423
419
EGF
TGFB
E2
TNF
IFNG
0
45
5
285
31
54
49
E2
Size of each list
419
209.5
0
361
389
419
TNF
IFNG
E2
TNF
IL1B
0
45
0
307
9
0
103
E2
Size of each list
419
209.5
0
361
352
419
TNF
IL1B
E2
TNF
PGF2a
43
2
4
282
34
56
47
E2
Size of each list
419
209.5
0
361
344
419
TNF
PGF2a
E2
TNF
LTD4
9
36
5
287
29
11
92
E2
Size of each list
419
209.5
0
361
339
419
TNF
LTD4
E2
TNF
CG complex
9
36
9
301
15
13
90
E2
Size of each list
419
209.5
0
361
359
419
TNF
CG complex
E2
TNF
IGF1
9
36
5
307
9
10
93
E2
Size of each list
419
209.5
0
361
358
419
TNF
IGF1
E2
TNF
Oleic acid
40
5
20
272
44
13
90
E2
Size of each list
419
209.5
0
361
310
419
TNF
Oleic acid
E2
TNF
Sphingosin
39
6
13
262
54
15
88
E2
Size of each list
419
209.5
0
361
296
419
TNF
Sphingosin
E2
TNF
Aldosterone
30
15
30
276
40
18
85
E2
Size of each list
419
209.5
0
361
339
419
TNF
Aldosterone
E2
Oleic acid
Aldosterone
21
4
41
264
21
30
104
E2
Size of each list
419
209.5
0
310
339
419
Oleic acid
Aldosterone
E2
IFNG
Aldosterone
34
16
29
268
71
26
54
E2
Size of each list
419
209.5
0
389
339
419
IFNG
Aldosterone
E2
Oleic acid
Sphingosin
13
12
7
268
17
9
125
E2
Size of each list
419
209.5
0
310
296
419
Oleic acid
Sphingosin
E2
Aldosterone
E2
IFNG
26
47
5
3
57
12
21
25
25
243
7
15
14
1
9
Size of each list
419
209.5
0
310
339
419
389
Oleic acid
Aldosterone
E2
IFNG

Figure S9

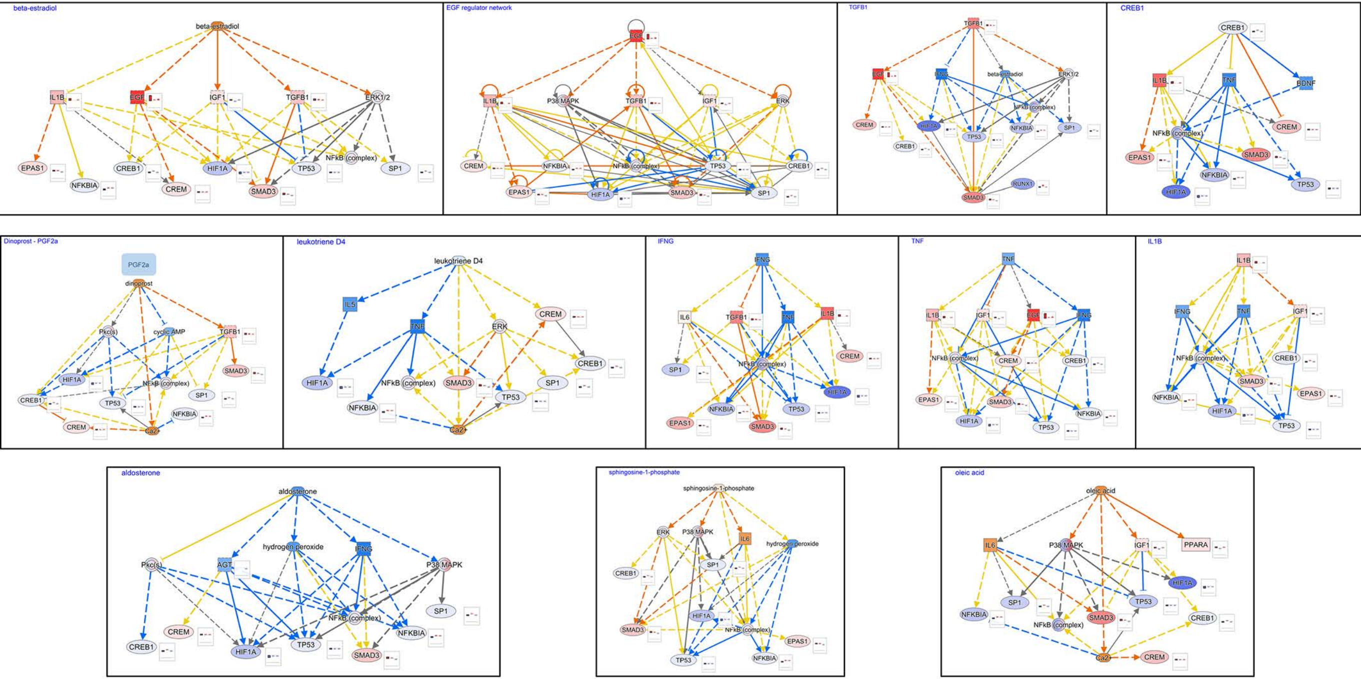

beta-estradiol
beta-estradiol
IL1B
EGF
IGF1
TGFB1
ERK1/2
EPAS1
NFKBIA
CREB1
CREM
HIF1A
SMAD3
TP53
NFkB (complex)
SP1
EGF regulator network
EGF
IL1B
P38 MAPK
TGFB1
IGF1
ERK
CREM
NFKBIA
NFkB (complex)
TP53
CREB1
EPAS1
HIF1A
SMAD3
SP1
TGFB1
TGFB1
EGF
IFNG
beta-estradiol
ERK1/2
CREM
HIF1A
TP53
NFkB (complex)
NFKBIA
SP1
CREB1
RUNX1
SMAD3
CREB1
CREB1
IL1B
TNF
BDNF
CREM
NFkB (complex)
EPAS1
SMAD3
NFKBIA
TP53
HIF1A
Dinoprost - PGF2a
PGF2a
dinoprost
Pkc(s)
cyclic AMP
TGFB1
SMAD3
HIF1A
NFkB (complex)
SP1
CREB1
TP53
NFKBIA
CREM
Ca2+
leukotriene D4
leukotriene D4
IL5
TNF
ERK
CREM
CREB1
HIF1A
NFkB (complex)
SMAD3
SP1
TP53
NFKBIA
Ca2+
IFNG
IFNG
IL6
TGFB1
TNF
IL1B
CREM
SP1
NFkB (complex)
HIF1A
NFKBIA
TP53
EPAS1
SMAD3
TNF
TNF
IL1B
IGF1
EGF
IFNG
NFkB (complex)
CREM
CREB1
EPAS1
SMAD3
HIF1A
TP53
NFKBIA
IL1B
IL1B
IFNG
TNF
IGF1
NFkB (complex)
CREB1
SMAD3
EPAS1
NFKBIA
HIF1A
TP53
aldosterone
aldosterone
hydrogen peroxide
IFNG
Pkc(s)
AGT
P38 MAPK
SP1
NFkB (complex)
CREM
NFKBIA
CREB1
HIF1A
TP53
SMAD3
sphingosine-1-phosphate
sphingosine-1-phosphate
ERK
P38 MAPK
IL6
hydrogen peroxide
SP1
CREB1
HIF1A
NFkB (complex)
SMAD3
EPAS1
TP53
NFKBIA
oleic acid
oleic acid
IL6
P38 MAPK
IGF1
PPARA
HIF1A
SP1
TP53
NFKBIA
SMAD3
CREB1
NFkB (complex)
Ca2+
CREM

Figure S10

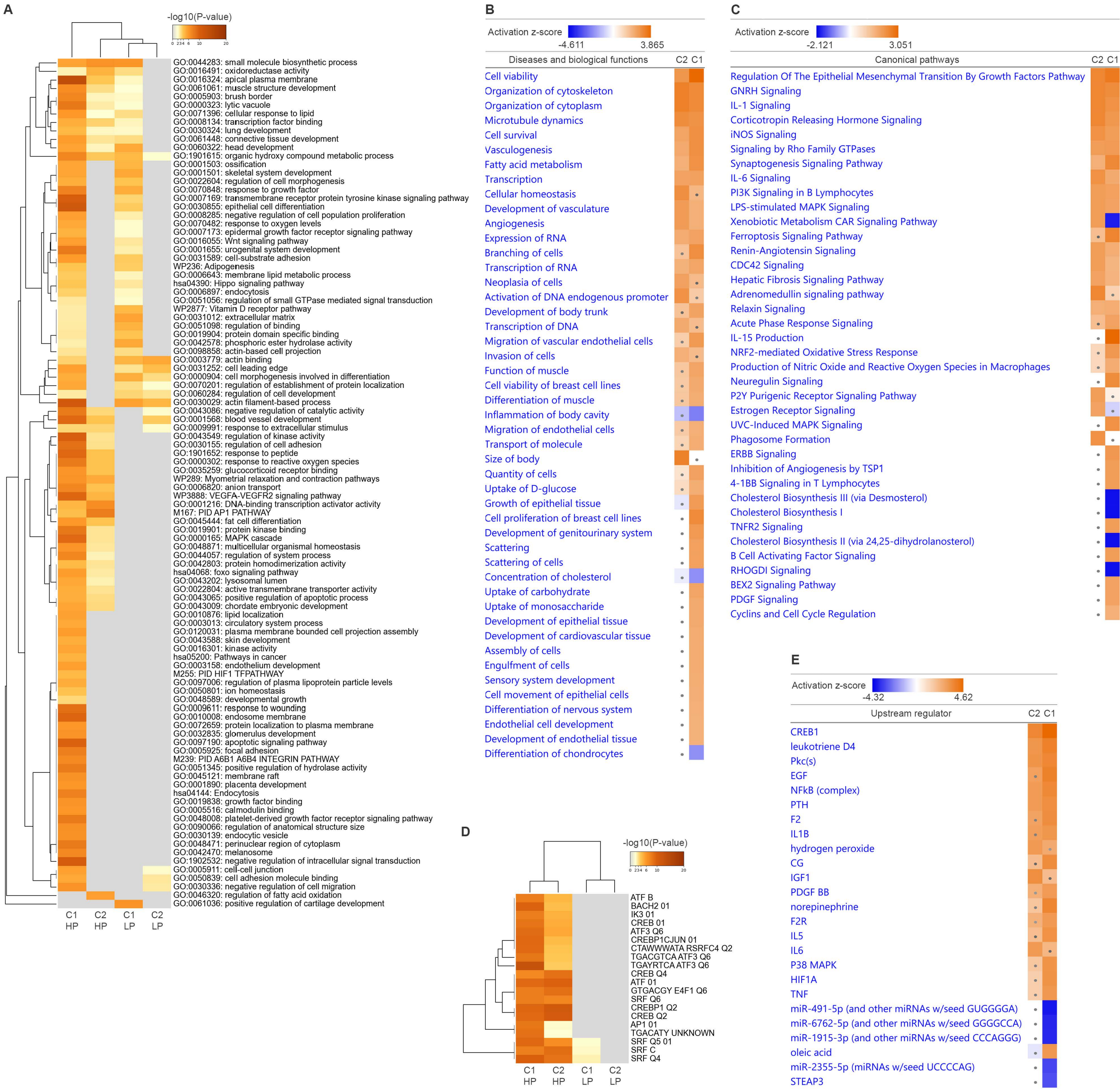
A
-log10(P-value)
0 2 3 4 6 10 20
GO:0044283: small molecule biosynthetic process
GO:0016491: oxidoreductase activity
GO:0016324: apical plasma membrane
GO:0061061: muscle structure development
GO:0005903: brush border
GO:0000323: lytic vacuole
GO:0071396: cellular response to lipid
GO:0008134: transcription factor binding
GO:0030324: lung development
GO:0061448: connective tissue development
GO:0060322: head development
GO:1901615: organic hydroxy compound metabolic process
GO:0001503: ossification
GO:0001501: skeletal system development
GO:0022604: regulation of cell morphogenesis
GO:0070848: response to growth factor
GO:0007169: transmembrane receptor protein tyrosine kinase signaling pathway
GO:0030855: epithelial cell differentiation
GO:0008285: negative regulation of cell population proliferation
GO:0070482: response to oxygen levels
GO:0007173: epidermal growth factor receptor signaling pathway
GO:0016055: Wnt signaling pathway
GO:0001655: urogenital system development
GO:0031589: cell-substrate adhesion
WP236: Adipogenesis
GO:0006643: membrane lipid metabolic process
hsa04390: Hippo signaling pathway
GO:0006897: endocytosis
GO:0051056: regulation of small GTPase mediated signal transduction
WP2877: Vitamin D receptor pathway
GO:0031012: extracellular matrix
GO:0051098: regulation of binding
GO:0019904: protein domain specific binding
GO:0042578: phosphoric ester hydrolase activity
GO:0098858: actin-based cell projection
GO:0003779: actin binding
GO:0031252: cell leading edge
GO:0000904: cell morphogenesis involved in differentiation
GO:0070201: regulation of establishment of protein localization
GO:0060284: regulation of cell development
GO:0030029: actin filament-based process
GO:0043086: negative regulation of catalytic activity
GO:0001568: blood vessel development
GO:0009991: response to extracellular stimulus
GO:0043549: regulation of kinase activity
GO:0030155: regulation of cell adhesion
GO:1901652: response to peptide
GO:0000302: response to reactive oxygen species
GO:0035259: glucocorticoid receptor binding
WP289: Myometrial relaxation and contraction pathways
GO:0006820: anion transport
WP3888: VEGFA-VEGFR2 signaling pathway
GO:0001216: DNA-binding transcription activator activity
M167: PID AP1 PATHWAY
GO:0045444: fat cell differentiation
GO:0019901: protein kinase binding
GO:0000165: MAPK cascade
GO:0048871: multicellular organismal homeostasis
GO:0044057: regulation of system process
GO:0042803: protein homodimerization activity
hsa04068: foxo signaling pathway
GO:0043202: lysosomal lumen
GO:0022804: active transmembrane transporter activity
GO:0043065: positive regulation of apoptotic process
GO:0043009: chordate embryonic development
GO:0010876: lipid localization
GO:0003013: circulatory system process
GO:0120031: plasma membrane bounded cell projection assembly
GO:0043588: skin development
GO:0016301: kinase activity
hsa05200: Pathways in cancer
GO:0003158: endothelium development
M255: PID HIF1 TFPATHWAY
GO:0097006: regulation of plasma lipoprotein particle levels
GO:0050801: ion homeostasis
GO:0048589: developmental growth
GO:0009611: response to wounding
GO:0010008: endosome membrane
GO:0072659: protein localization to plasma membrane
GO:0032835: glomerulus development
GO:0097190: apoptotic signaling pathway
GO:0005925: focal adhesion
M239: PID A6B1 A6B4 INTEGRIN PATHWAY
GO:0051345: positive regulation of hydrolase activity
GO:0045121: membrane raft
GO:0001890: placenta development
hsa04144: Endocytosis
GO:0019838: growth factor binding
GO:0005516: calmodulin binding
GO:0048008: platelet-derived growth factor receptor signaling pathway
GO:0090066: regulation of anatomical structure size
GO:0030139: endocytic vesicle
GO:0048471: perinuclear region of cytoplasm
GO:0042470: melanosome
GO:1902532: negative regulation of intracellular signal transduction
GO:0005911: cell-cell junction
GO:0050839: cell adhesion molecule binding
GO:0030336: negative regulation of cell migration
GO:0046320: regulation of fatty acid oxidation
GO:0061036: positive regulation of cartilage development
C1 HP
C2 HP
C1 LP
C2 LP
B
Activation z-score
-4.611
3.865
Diseases and biological functions
C2 C1
Cell viability
Organization of cytoskeleton
Organization of cytoplasm
Microtubule dynamics
Cell survival
Vasculogenesis
Fatty acid metabolism
Transcription
Cellular homeostasis
Development of vasculature
Angiogenesis
Expression of RNA
Branching of cells
Transcription of RNA
Neoplasia of cells
Activation of DNA endogenous promoter
Development of body trunk
Transcription of DNA
Migration of vascular endothelial cells
Invasion of cells
Function of muscle
Cell viability of breast cell lines
Differentiation of muscle
Inflammation of body cavity
Migration of endothelial cells
Transport of molecule
Size of body
Quantity of cells
Uptake of D-glucose
Growth of epithelial tissue
Cell proliferation of breast cell lines
Development of genitourinary system
Scattering
Scattering of cells
Concentration of cholesterol
Uptake of carbohydrate
Uptake of monosaccharide
Development of epithelial tissue
Development of cardiovascular tissue
Assembly of cells
Engulfment of cells
Sensory system development
Cell movement of epithelial cells
Differentiation of nervous system
Endothelial cell development
Development of endothelial tissue
Differentiation of chondrocytes
C
Activation z-score
-2.121
3.051
Canonical pathways
C2 C1
Regulation Of The Epithelial Mesenchymal Transition By Growth Factors Pathway
GNRH Signaling
IL-1 Signaling
Corticotropin Releasing Hormone Signaling
iNOS Signaling
Signaling by Rho Family GTPases
Synaptogenesis Signaling Pathway
IL-6 Signaling
PI3K Signaling in B Lymphocytes
LPS-stimulated MAPK Signaling
Xenobiotic Metabolism CAR Signaling Pathway
Ferroptosis Signaling Pathway
Renin-Angiotensin Signaling
CDC42 Signaling
Hepatic Fibrosis Signaling Pathway
Adrenomedullin signaling pathway
Relaxin Signaling
Acute Phase Response Signaling
IL-15 Production
NRF2-mediated Oxidative Stress Response
Production of Nitric Oxide and Reactive Oxygen Species in Macrophages
Neuregulin Signaling
P2Y Purigenic Receptor Signaling Pathway
Estrogen Receptor Signaling
UVC-Induced MAPK Signaling
Phagosome Formation
ERBB Signaling
Inhibition of Angiogenesis by TSP1
4-1BB Signaling in T Lymphocytes
Cholesterol Biosynthesis III (via Desmosterol)
Cholesterol Biosynthesis I
TNFR2 Signaling
Cholesterol Biosynthesis II (via 24,25-dihydrolanosterol)
B Cell Activating Factor Signaling
RHOGDI Signaling
BEX2 Signaling Pathway
PDGF Signaling
Cyclins and Cell Cycle Regulation
D
-log10(P-value)
0 2 3 4 6 10 20
ATF B
BACH2 01
IK3 01
CREB 01
ATF3 Q6
CREBP1CJUN 01
CTAWWWATA RSRFC4 Q2
TGACGTCA ATF3 Q6
TGAYRTCA ATF3 Q6
CREB Q4
ATF 01
GTGACGY E4F1 Q6
SRF Q6
CREBP1 Q2
CREB Q2
AP1 01
TGACATY UNKNOWN
SRF Q5 01
SRF C
SRF Q4
C1 HP
C2 HP
C1 LP
C2 LP
E
Activation z-score
-4.32
4.62
Upstream regulator
C2 C1
CREB1
leukotriene D4
Pkc(s)
EGF
NFkB (complex)
PTH
F2
IL1B
hydrogen peroxide
CG
IGF1
PDGF BB
norepinephrine
F2R
IL5
IL6
P38 MAPK
HIF1A
TNF
miR-491-5p (and other miRNAs w/seed GUGGGGA)
miR-6762-5p (and other miRNAs w/seed GGGGCCA)
miR-1915-3p (and other miRNAs w/seed CCCAGGG)
oleic acid
miR-2355-5p (miRNAs w/seed UCCCCAG)
STEAP3

Figure S11

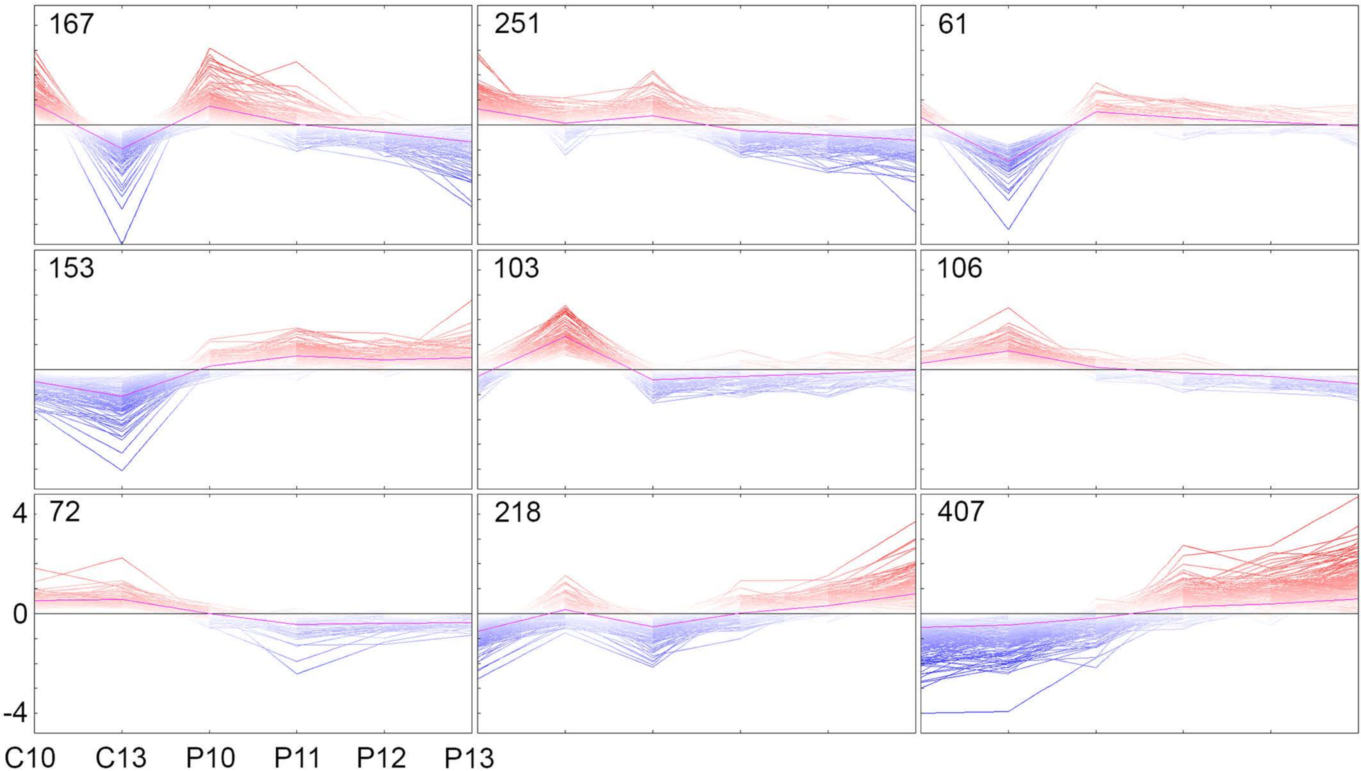

Figure S12

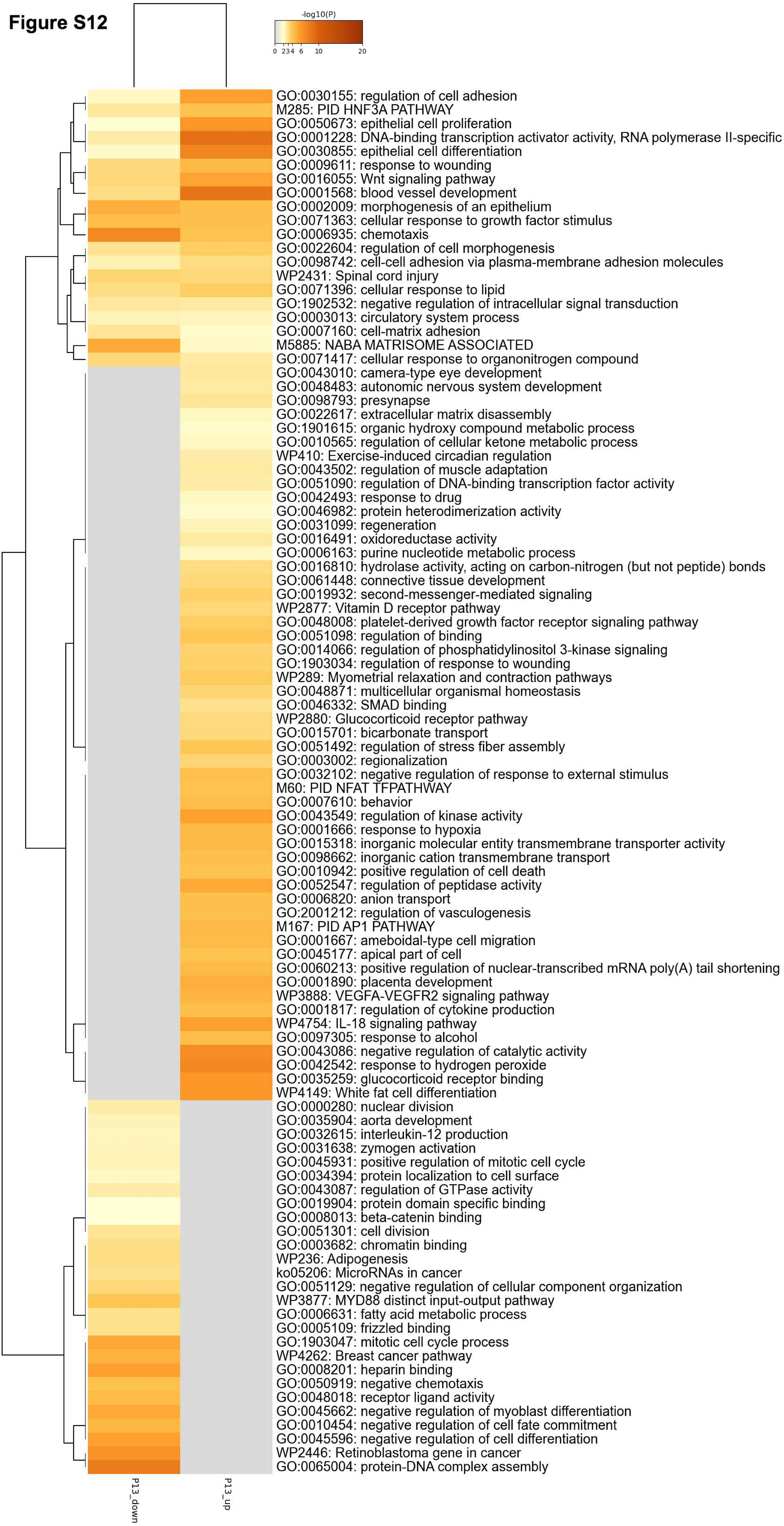
-log10(P)
0 2 3 4 6 10 20
GO:0030155: regulation of cell adhesion
M285: PID HNF3A PATHWAY
GO:0050673: epithelial cell proliferation
GO:0001228: DNA-binding transcription activator activity, RNA polymerase II-specific
GO:0030855: epithelial cell differentiation
GO:0009611: response to wounding
GO:0016055: Wnt signaling pathway
GO:0001568: blood vessel development
GO:0002009: morphogenesis of an epithelium
GO:0071363: cellular response to growth factor stimulus
GO:0006935: chemotaxis
GO:0022604: regulation of cell morphogenesis
GO:0098742: cell-cell adhesion via plasma-membrane adhesion molecules
WP2431: Spinal cord injury
GO:0071396: cellular response to lipid
GO:1902532: negative regulation of intracellular signal transduction
GO:0003013: circulatory system process
GO:0007160: cell-matrix adhesion
M5885: NABA MATRISOME ASSOCIATED
GO:0071417: cellular response to organonitrogen compound
GO:0043010: camera-type eye development
GO:0048483: autonomic nervous system development
GO:0098793: presynapse
GO:0022617: extracellular matrix disassembly
GO:1901615: organic hydroxy compound metabolic process
GO:0010565: regulation of cellular ketone metabolic process
WP410: Exercise-induced circadian regulation
GO:0043502: regulation of muscle adaptation
GO:0051090: regulation of DNA-binding transcription factor activity
GO:0042493: response to drug
GO:0046982: protein heterodimerization activity
GO:0031099: regeneration
GO:0016491: oxidoreductase activity
GO:0006163: purine nucleotide metabolic process
GO:0016810: hydrolase activity, acting on carbon-nitrogen (but not peptide) bonds
GO:0061448: connective tissue development
GO:0019932: second-messenger-mediated signaling
WP2877: Vitamin D receptor pathway
GO:0048008: platelet-derived growth factor receptor signaling pathway
GO:0051098: regulation of binding
GO:0014066: regulation of phosphatidylinositol 3-kinase signaling
GO:1903034: regulation of response to wounding
WP289: Myometrial relaxation and contraction pathways
GO:0048871: multicellular organismal homeostasis
GO:0046332: SMAD binding
WP2880: Glucocorticoid receptor pathway
GO:0015701: bicarbonate transport
GO:0051492: regulation of stress fiber assembly
GO:0003002: regionalization
GO:0032102: negative regulation of response to external stimulus
M60: PID NFAT TFPATHWAY
GO:0007610: behavior
GO:0043549: regulation of kinase activity
GO:0001666: response to hypoxia
GO:0015318: inorganic molecular entity transmembrane transporter activity
GO:0098662: inorganic cation transmembrane transport
GO:0010942: positive regulation of cell death
GO:0052547: regulation of peptidase activity
GO:0006820: anion transport
GO:2001212: regulation of vasculogenesis
M167: PID AP1 PATHWAY
GO:0001667: ameboidal-type cell migration
GO:0045177: apical part of cell
GO:0060213: positive regulation of nuclear-transcribed mRNA poly(A) tail shortening
GO:0001890: placenta development
WP3888: VEGFA-VEGFR2 signaling pathway
GO:0001817: regulation of cytokine production
WP4754: IL-18 signaling pathway
GO:0097305: response to alcohol
GO:0043086: negative regulation of catalytic activity
GO:0042542: response to hydrogen peroxide
GO:0035259: glucocorticoid receptor binding
WP4149: White fat cell differentiation
GO:0000280: nuclear division
GO:0035904: aorta development
GO:0032615: interleukin-12 production
GO:0031638: zymogen activation
GO:0045931: positive regulation of mitotic cell cycle
GO:0034394: protein localization to cell surface
GO:0043087: regulation of GTPase activity
GO:0019904: protein domain specific binding
GO:0008013: beta-catenin binding
GO:0051301: cell division
GO:0003682: chromatin binding
WP236: Adipogenesis
ko05206: MicroRNAs in cancer
GO:0051129: negative regulation of cellular component organization
WP3877: MYD88 distinct input-output pathway
GO:0006631: fatty acid metabolic process
GO:0005109: frizzled binding
GO:1903047: mitotic cell cycle process
WP4262: Breast cancer pathway
GO:0008201: heparin binding
GO:0050919: negative chemotaxis
GO:0048018: receptor ligand activity
GO:0045662: negative regulation of myoblast differentiation
GO:0010454: negative regulation of cell fate commitment
GO:0045596: negative regulation of cell differentiation
WP2446: Retinoblastoma gene in cancer
GO:0065004: protein-DNA complex assembly
P13_down
P13_up

Figure S13

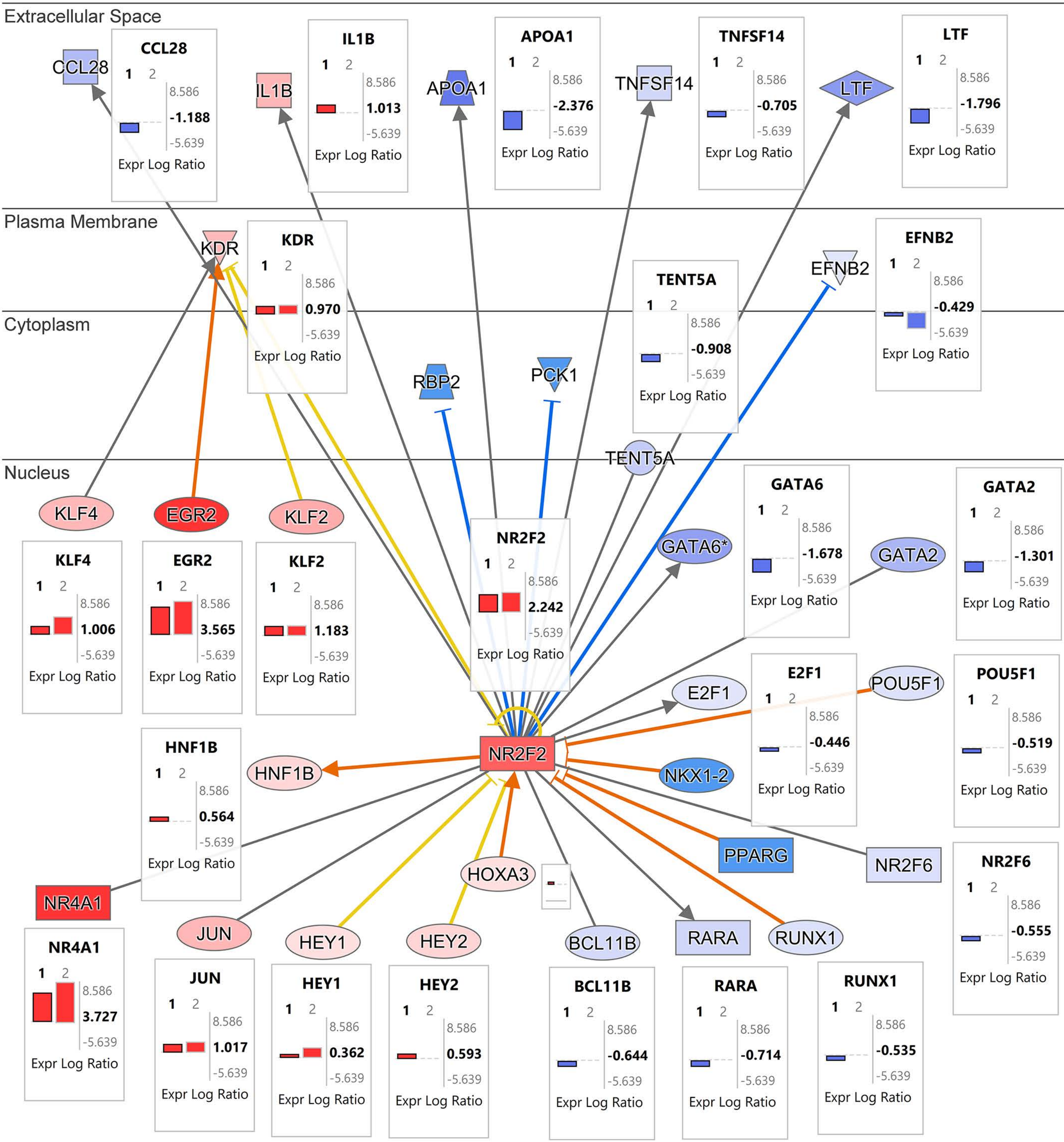
Extracellular Space
Plasma Membrane
Cytoplasm
Nucleus
CCL28 1 2 8.586 -1.188 -5.639 Expr Log Ratio
IL1B 1 2 8.586 1.013 -5.639 Expr Log Ratio
APOA1 1 2 8.586 -2.376 -5.639 Expr Log Ratio
TNFSF14 1 2 8.586 -0.705 -5.639 Expr Log Ratio
LTF 1 2 8.586 -1.796 -5.639 Expr Log Ratio
KDR 1 2 8.586 0.970 -5.639 Expr Log Ratio
TENT5A 1 2 8.586 -0.908 -5.639 Expr Log Ratio
EFNB2 1 2 8.586 -0.429 -5.639 Expr Log Ratio
RBP2
PCK1
KLF4 1 2 8.586 1.006 -5.639 Expr Log Ratio
EGR2 1 2 8.586 3.565 -5.639 Expr Log Ratio
KLF2 1 2 8.586 1.183 -5.639 Expr Log Ratio
NR2F2 1 2 8.586 2.242 -5.639 Expr Log Ratio
GATA6*
GATA6 1 2 8.586 -1.678 -5.639 Expr Log Ratio
GATA2 1 2 8.586 -1.301 -5.639 Expr Log Ratio
E2F1 1 2 8.586 -0.446 -5.639 Expr Log Ratio
POU5F1 1 2 8.586 -0.519 -5.639 Expr Log Ratio
HNF1B 1 2 8.586 0.564 -5.639 Expr Log Ratio
NKX1-2
PPARG
NR2F6
HOXA3
NR4A1
JUN
HEY1
HEY2
BCL11B
RARA
RUNX1
NR2F6 1 2 8.586 -0.555 -5.639 Expr Log Ratio
NR4A1 1 2 8.586 3.727 -5.639 Expr Log Ratio
JUN 1 2 8.586 1.017 -5.639 Expr Log Ratio
HEY1 1 2 8.586 0.362 -5.639 Expr Log Ratio
HEY2 1 2 8.586 0.593 -5.639 Expr Log Ratio
BCL11B 1 2 8.586 -0.644 -5.639 Expr Log Ratio
RARA 1 2 8.586 -0.714 -5.639 Expr Log Ratio
RUNX1 1 2 8.586 -0.535 -5.639 Expr Log Ratio
