# Supplementary material for: Spatiotemporal endometrial transcriptome analysis revealed the luminal epithelium as key player during initial maternal recognition of pregnancy in the mare
Source: Sci Rep. 2021 Nov 16;11:22293. doi: 10.1038/s41598-021-01785-3 (PMC8595723; doi:10.1038/s41598-021-01785-3)
Supplement: Supplementary file 3 — Supplementary Table S1. [file 41598_2021_1785_MOESM3_ESM.docx]

Supplemental Table 1. Samples collected for pregnancy and control groups.

|  | Day of pregnancy | | | | Day of estrous cycle | |
| --- | --- | --- | --- | --- | --- | --- |
| Mare ID | 10 | 11 | 12 | 13 | 10 | 13 |
| 10 | X |  |  |  | X |  |
| 11 | X | X |  |  |  |  |
| 12 |  | X |  | X |  |  |
| 13 |  | X |  |  |  |  |
| 15 |  |  |  | X | X | X |
| 16 |  |  | X |  | X | X |
| 17 | X | X | X | X |  | X |
| 18 | X |  |  |  |  |  |
| 20 |  |  | X |  | X |  |
| 20 |  |  | X |  |  |  |
| 21 |  |  |  | X | X | X |
| 23 | X | X | X | X |  | X |
