# Supplementary material for: Spatiotemporal endometrial transcriptome analysis revealed the luminal epithelium as key player during initial maternal recognition of pregnancy in the mare
Source: Sci Rep. 2021 Nov 16;11:22293. doi: 10.1038/s41598-021-01785-3 (PMC8595723; doi:10.1038/s41598-021-01785-3)
Supplement: Supplementary file 4 — Supplementary Table S2. [file 41598_2021_1785_MOESM4_ESM.docx]

Supplemental Table 2: Size and number of recovered conceptuses.

| Mare ID | Pregnancy day | Embryo | Size in ultrasonography [mm] | Size measured after collection [mm] |
| --- | --- | --- | --- | --- |
| 10 | 10 | 1 | nd | 3 |
|  |  | 2 | nd | 4 |
| 11 | 10 | 1 | nd | 4 |
| 17 | 10 | 1 | 5 | 5 |
| 18 | 10 | 1 | nd | 3 |
| 23 | 10 | 1 | nd | 4 |
| 11 | 11 | 1 | 8 | 10 |
| 12 | 11 | 1 | nd | 3 |
| 13 | 11 | 1 | nd | 7 |
|  |  | 2 | nd | 7 |
| 17 | 11 | 1 | 7.6 | 9 |
| 23 | 11 | 1 | 5.7 | 8 |
| 16 | 12 | 1 | 7.3*6.8 | 7 |
| 17 | 12 | 1 | 9.7*9.3 | 10 |
|  |  | 2 | 9.6*9.8 | 11 |
| 20 | 12 | 1 | nd | 4 |
| 20 | 12 | 1 | 9.2*8.8 | 9 |
| 23 | 12 | 1 | 10.6*9.6 | nd |
| 12 | 13 | 1 | 10.2*10.5 | 15 |
| 15 | 13 | 1 | nd | 20 |
|  |  | 2 | nd | 26 |
| 17 | 13 | 1 | nd | nd |
|  |  | 2 | nd | nd |
| 21 | 13 | 1 | 9.7*8.5 | 11 |
| 23 | 13 | 1 | nd | 8 |
