# Supplementary material for: Spatiotemporal endometrial transcriptome analysis revealed the luminal epithelium as key player during initial maternal recognition of pregnancy in the mare
Source: Sci Rep. 2021 Nov 16;11:22293. doi: 10.1038/s41598-021-01785-3 (PMC8595723; doi:10.1038/s41598-021-01785-3)
Supplement: Supplementary file 5 — Supplementary Table S3. [file 41598_2021_1785_MOESM5_ESM.docx]

Supplemental Table 3: Breed, age, and endometrial category in the scale of Kenney and Doig (1986).

| #Mare ID | Breed | Age | Kenney and Doig |
| --- | --- | --- | --- |
| 10 | Thoroughbred | 14 | IIA |
| 11 | Arabian | 13 | IIA (to IIB) |
| 12 | Tennessee Walker | 7 | I |
| 13 | Mixed | 8 | I (to IIA) |
| 15 | Quarterhorse | 4 | I |
| 16 | Thoroughbred | 15 | IIB |
| 17 | Tennessee Walker | 9 | IIA |
| 18 | Quarterhorse | 9 | I (to IIA) |
| 20 | Arabian | 11 | I |
| 21 | Standardbred | 8 | I (to IIA) |
| 23 | Standardbred | 14 | I (to IIA) |
